# Gene Set Analysis: Challenges, Opportunities, and Future Research

**DOI:** 10.3389/fgene.2020.00654

**Published:** 2020-06-30

**Authors:** Farhad Maleki, Katie Ovens, Daniel J. Hogan, Anthony J. Kusalik

**Affiliations:** Department of Computer Science, University of Saskatchewan, Saskatoon, SK, Canada

**Keywords:** gene expression, gene set analysis, gene set enrichment, gene set database, sensitivity, specificity

## Abstract

Gene set analysis methods are widely used to provide insight into high-throughput gene expression data. There are many gene set analysis methods available. These methods rely on various assumptions and have different requirements, strengths and weaknesses. In this paper, we classify gene set analysis methods based on their components, describe the underlying requirements and assumptions for each class, and provide directions for future research in developing and evaluating gene set analysis methods.

## 1. Introduction

High-throughput technologies such as DNA microarrays and RNA-Seq are widely used to monitor the activity of thousands of genes in a single experiment. The primary challenge to realizing the potential of these technologies is gaining biological insight from the generated data.

The early approach for analysing gene expression data was single-gene analysis, where expression measures of each gene for case and control samples are compared using a statistical test such as *t*-test or Wilcoxon rank-sum test and a *p*-value is calculated. Then, in order to reduce the number of false positives resulting from multiple comparisons, an adjustment for multiple comparison is made. Next, genes with a adjusted *p*-value smaller than a given threshold are predicted as being differentially expressed. Finally, a biological interpretation is attempted using these genes. This approach suffers from several shortcomings:

- In a high-throughput gene expression study, many single-gene tests are typically performed. Consequently, adjustment for multiple comparisons is performed for a large number of genes. Such adjustments may lead to many false negatives by detecting very few or even no gene as being differentially expressed (Sreekumar et al., 2002; Yang et al., 2002; Mootha et al., 2003). This issue is more pronounced when using conservative methods, such as Bonferroni and Šídák for multiple comparison adjustment (Drăghici, 2016).
- In the single-gene approach often researchers use arbitrary cutoff values to choose a reasonable number of genes for further study and interpretation. Different choices of threshold value may lead to different biological interpretations (Pan et al., 2005). Conservative threshold values may cause false negatives and relaxed thresholds may cause false positives (Breslin et al., 2004; Ben-Shaul et al., 2005).
- Cellular processes are often associated with changes in the expression patterns of groups of genes that share common biological functions or attributes. A meaningful change in a group of these genes is more biologically reliable and interpretable than a change in a single gene. *A priori* knowledge about some of these sets of genes is available through public online databases such as GO (Consortium et al., 2015), KEGG (Kanehisa et al., 2015), and OMIM (Amberger et al., 2009). The single-gene approach disregards this information. Incorporating this information in the data analysis may provide valuable insight about underlying biological processes or functions.
- Although high-throughput technologies make the monitoring of expression of thousands of genes in a single experiment possible, they introduce a challenge of dealing with high dimensional data, often referred to as the “curse of dimensionality” (Berrar et al., 2003). To deal with high dimensional data, dimensionality reduction methods are used for downstream analyses and visualizations. Relying on sets of biologically related genes is the most intuitive and biologically relevant approach to dimensionality reduction in high-throughput gene expression studies.
- When differences in measured values for a single-gene across treatments are subtle, the single-gene approach makes it difficult to differentiate the true difference in gene expression from the difference due to biological variability of samples (Mootha et al., 2003; Subramanian et al., 2005). Gene set analysis, on the other hand, might be able to detect such subtle but concordant changes in expression pattern of genes within a gene set.
- Multi-functional genes, i.e., genes that are involved in multiple biological activities, are commonplace. For example, Pritykin et al. (2015) reported that multi-functional genes make up 24, 26, and 19% of annotated genes in *Drosophila melanogaster, Homo sapiens*, and *Saccharomyces cerevisiae*, respectively. The presence of such a large number of multi-functional genes means single-gene analysis may lead to false or ambiguous conclusions.
- Single-gene approach may report several hundred to a few thousand genes as being differentially expressed. Interpreting a long list of differentially expressed genes is a cumbersome task prone to investigator bias toward a hypothesis of interest.

Gene set analysis, also know as enrichment analysis, is an attempt to resolve these shortcomings and to gain insight from gene expression data. The primary aim of gene set analysis is to identify enrichment or depletion of expression levels of a given set of genes of interest, referred to as a gene set. In this paper, we use the phrase “differentially enriched” to describe gene sets that either are enriched (more expression activity) or depleted (less expression activity).

Gene sets are defined based on various criteria such as membership in certain biological pathways or being co-expressed together under a certain condition. These gene sets are gathered into collections known as gene set databases. MSigDB (Subramanian et al., 2005), GeneSigDB (Culhane et al., 2011), and GeneSetDB (Araki et al., 2012) are three gene set collections specifically developed for gene set analysis. These collections of gene sets allow researchers to analyse the activity of groups of biologically related genes rather than single genes to determine which of these groups are relevant to a phenotype of interest. The phenotypes of interest should be two different conditions, e.g., healthy vs. diseased, or a specific treatment versus no treatment. There are a large number of gene set analysis methods available (Huang et al., 2009; Mitrea et al., 2013), which have been used for a variety applications, including studying complex diseases (Suárez-Fariñas et al., 2010; Wu et al., 2016; Noori et al., 2020), drug responses Bateman et al. (2014), and developmental stages across species (Cardoso-Moreira et al., 2019) (See Table S1). These methods differ in their various components such as their underlying assumptions, notion of enrichment, null hypotheses, and significance assessment procedures. Study of gene set analysis methods based on their components helps to understand the strengths and weaknesses of each category of methods, select an appropriate method for a given experiment, facilitate the interpretation of the outcomes of the analysis, and develop new methods with higher sensitivity and specificity.

Although we provide a list of more than 100 gene set analysis methods/tools (see Supplementary Materials), the purpose of this review is not to discuss all tools available for gene set analysis. Rather, using a representative set of methods, we aim to provide a modular overview of gene set analysis methods based on their various components. We highlight the shortcomings of each class of methods and the challenges they face.

The rest of the paper is organized as follows. In section 2, we survey most widely used over-representation analysis (ORA) and functional class scoring (FCS) methods. Different significance assessment approaches and null hypotheses are covered in sections 3 and 4, respectively. Pathway topology-based methods are briefly surveyed in section 5. Section 6 describes the challenges facing gene set analysis methods. In section 7, we provide directions for future research in developing and evaluating gene set analysis methods. Finally, section 8 concludes the paper with a short summary.

## 2. Gene Set Analysis

**Figure 1:**
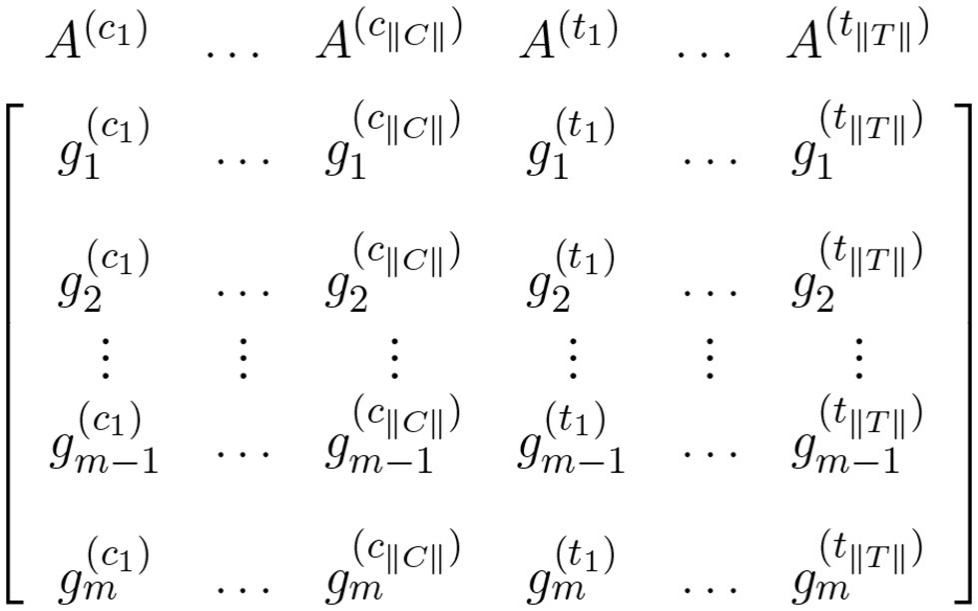
Expression matrix for a pairwise comparison where $A^{\left(c_{1}\right)},\ldots ,A^{\left(c_{\left||C|\right|}\right)}$ columns represent control samples and $A^{\left(t_{1}\right)},\ldots ,A^{\left(t_{\left||T|\right|}\right)}$ columns represent case samples. In this figure, $g_{i}^{\left(c_{j}\right)}$ and $g_{i}^{\left(t_{j}\right)}$ represent the expression measures for the *i*^*th*^ gene in the ${c_{j}}^{th}$ control sample and ${t_{j}}^{th}$ case sample, respectively.

Data from a high-throughput case-control experiment can be organized in an expression matrix. This matrix is generated by joining the corresponding expression values for all samples in the experiment. Each column of the matrix corresponds to the expression measures for one sample and each row corresponds to the expression measures for one gene across all samples. This expression matrix is the input for expression analyses including single-gene and gene set analysis. Figure 1 shows an expression matrix with ||*C*|| control samples and ||*T*|| case samples.

**Figure 2:**
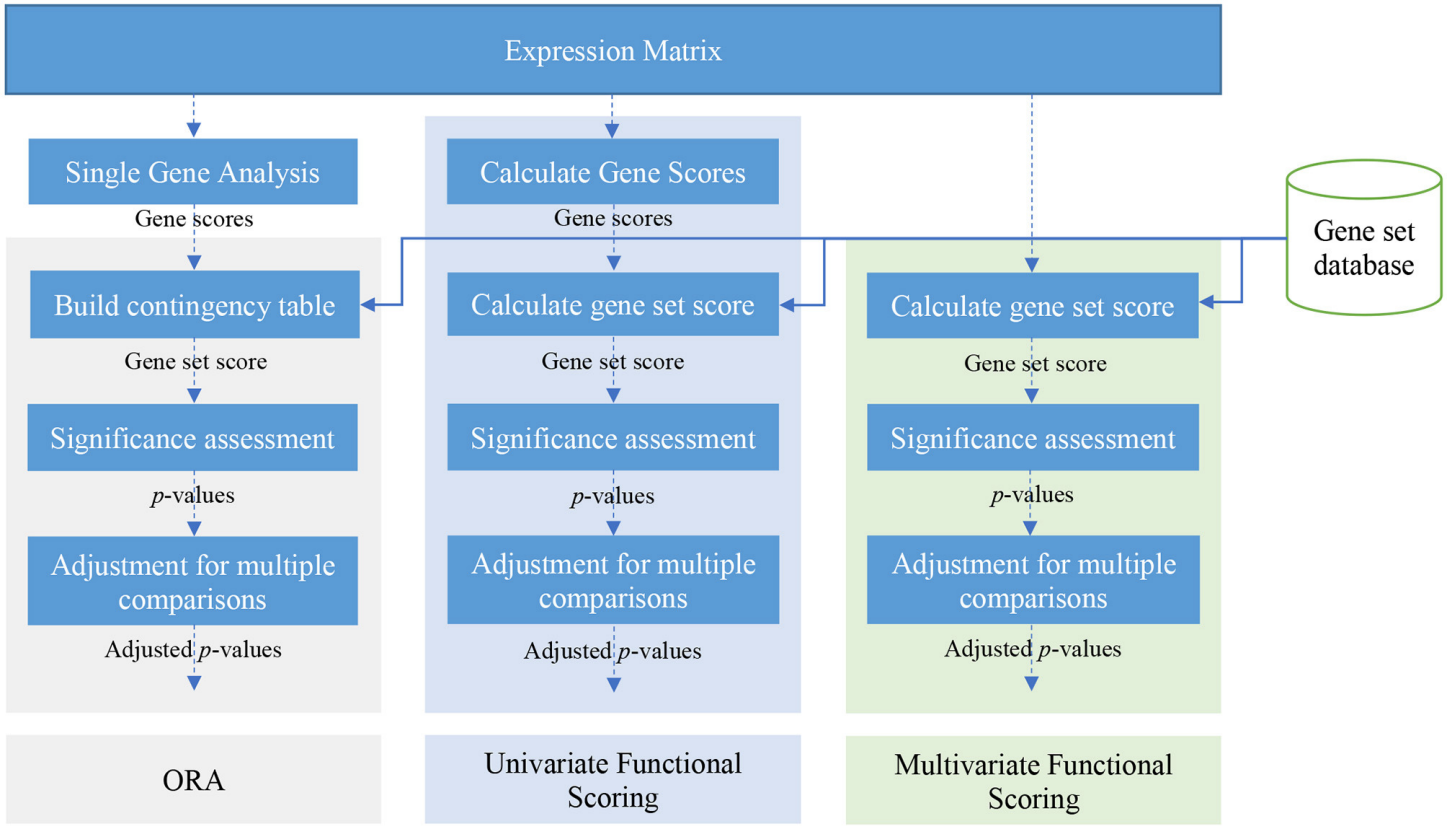
A schematic view of over-representation analysis (ORA) and univariate and multivariate FCS methods.

There are many gene set analysis methods available. Over-representation analysis, functional class scoring, and pathway topology-based methods are three main categories of gene set analysis methods (Khatri et al., 2012). Figure 2 illustrates a schematic view of univariate and multivariate FCS methods and also ORA methods. In this paper, we focus on ORA and FCS methods that comprise the main body of gene set analysis methods used by researchers (the rest of section 2). We briefly discuss Pathway topology-based methods in section 5. For a comprehensive review and a comparison of topology-based methods see works by Mitrea et al. (2013) and Ihnatova et al. (2018).

### 2.1. Over-Representation Analysis

ORA is the natural extension of single-gene analysis and one of the most widely used classes of gene set analysis methods. Due to its simplicity, well-established underlying statistical model, and ease of implementation, ORA is available through many tools. Huang et al. (2009) listed 68 gene set analysis methods and tools of which 40 are ORA-based. These tools differ in their various components such as gene set database, data visualization, and user interface (Huang et al., 2009). ORA uses a list *L* of genes each predicted as being differentially expressed by a single-gene analysis method.

Given *L* and a gene set *G*_*i*_ that has ${n^{\prime }}_{i}$ genes in common with *L*, ORA considers *G*_*i*_ as being differentially enriched if the occurrence of ${n^{\prime }}_{i}$ differentially expressed genes in *G*_*i*_ is unlikely to be due to chance. Table 1 illustrates the contingency table representation for the over-representation of differentially expressed genes in *G*_*i*_ given *L* and *U*, where $\bar{G_{i}}$ is the set of all genes under study that are not members of *G*_*i*_. The set of *n* genes under study is called the reference set or background set and depicted by *U*, and $\bar{G_{i}}$ is the complement of *G*_*i*_ with respect to *U*.

**Table 1:** Representation of ORA as a contingency table.

	**Genes in L**	**Genes not in L**	**Total**
Genes in *G*_*i*_	$n_{i}\prime$	$\|G_{i}\|-n_{i}\prime$	||*G*_*i*_||
Genes in $\bar{G_{i}}$	$\|L\|-n_{i}\prime$	$n-\|G_{i}\|-(\left\|L\|-n_{i}\prime \right)$	*n* − ||*G*_*i*_||
Total	||*L*||	*n* − ||*L*||	*n*

*Each cell contains a count of genes satisfying the conditions associated with the row and column*.

Under the null hypothesis that there is no association between differential expression and membership in *G*_*i*_, we can assume that *G*_*i*_ is the result of a simple random sampling of ||*G*_*i*_|| genes from *U*; therefore, the probability of having ${n^{\prime }}_{i}$ differentially expressed genes within *G*_*i*_ can be calculated using the hypergeometric distribution as follows (Drăghici, 2016):

(1) f(ni′;n,‖Gi‖,‖L‖)=(‖Gi‖ni′)×(n-‖Gi‖‖L‖-ni′)(n‖L‖)

The significance of the association between genes in *G*_*i*_ and genes in *L* can be assessed using Fisher's exact test, as follows:

(2) $$\begin{array}{l} p=\sum_{j=n_{i}\prime }^{\left\|G_{i}\right\|}f\left(j;n,\|G_{i}\|,\|L\|\right)=1-\sum_{j=0}^{n_{i}\prime -1}f\left(j;n,\|G_{i}\|,\|L\|\right) \end{array}$$

Although Fisher's exact test gives the exact *p*-value for both small and large cell counts in Table 1, the calculation can become numerically unstable for large cell counts. Therefore, alternatives are also used to approximate the *p*-value.

For large values of *n*, the hypergeometric distribution tends to the binomial distribution. Therefore, the binomial distribution can be used to estimate the *p*-value for Fisher's exact test (Drăghici et al., 2003). The binomial estimation of Equation (1) is as follows:

(3) $$\begin{array}{l} f_{b}(n_{i}\prime ;\|L\|,\frac{\left\|G\right\|}{n})=\;\left(\frac{\left\|L\right\|}{n_{i}\prime }\right)\times {\left(\frac{\left\|G_{i}\right\|}{n}\right)}^{n_{i}\prime }\times {\left(1-\frac{\left\|G_{i}\right\|}{n}\right)}^{\|L\|-n_{i}\prime } \end{array}$$

Therefore, Equation (2) can be estimated as:

(4) $$\begin{array}{l} p=1-\overset{i-1}{\underset{j=0}{\sum }}f_{b}\left(j;\|L\|,\frac{\left\|G_{i}\right\|}{n}\right) \end{array}$$

where *f*_*b*_ in Equation (3) and (4) represents the binomial distribution density function.

Another alternative to estimate the *p*-value is the χ^2^ test for equality of proportions (Van Belle et al., 2004). This test has also been used in the context of over-representation analysis (Khatri et al., 2002; Drăghici et al., 2003; Zhong et al., 2004).

### 2.2. Functional Class Scoring Methods

The main assumptions of ORA are that genes are independent and equally effective in biological processes. Although these assumptions simplify problem modeling, they are not biologically valid. It is well-established that genes, proteins, and other biomolecules often act in concert (Tilford and Siemers, 2009). In addition, ORA only utilizes differentially expressed genes, which often are the result of applying a *p*-value cutoff, and all the quantitative measures for the rest of the genes are disregarded. However, a consistent change in the expression of genes—even those with a *p*-value slightly greater than the cutoff value—may contribute to the detection of pathway activities.

In contrast to ORA, the main goal of FCS methods is to use all information from an expression matrix to address the enrichment problem without relying on the aforementioned biologically invalid assumptions. Therefore, FCS methods—instead of working with a list of differentially expressed genes—take advantage of an expression matrix of gene expression measures for all genes to discern differential enrichment of gene sets.

There are many FCS methods available (see Table S1). These methods can be categorized into two classes: univariate and multivariate methods. In univariate FCS methods, usually a gene score is calculated for each gene using each row of the expression matrix. Then these gene scores are used to calculate a gene set score for each gene set. Finally, the significance of the gene set scores is assessed and differentially enriched gene sets are reported. Multivariate methods skip the step for calculating gene scores and directly calculate gene set scores from the expression matrix.

An FCS method often consists of a set of common components such as a gene score that is a statistic summarizing the expression level of a gene across control and case samples, a gene set score that summarizes the expression level of genes within a gene set as a single statistic, a procedure for significance assessment, and an adjustment for multiple comparisons.

#### 2.2.1. Univariate Functional Class Scoring Methods

GSEA (Mootha et al., 2003) is one of the most widely used univariate FCS methods. It uses a signal-to-noise ratio (SNR) difference between gene expression measures in control and case samples to calculate a gene score. The signal-to-noise ratio difference is as follows (Tamayo et al., 2016):

(5) $$\begin{array}{l} SNR(g_{i})=\;\frac{\frac{\sum_{j=1}^{\left||C|\right|}g_{i}^{\left(c_{j}\right)}}{\left||C|\right|}-\frac{\sum_{j=1}^{\left||T|\right|}g_{i}^{\left(t_{j}\right)}}{\left||T|\right|}}{{\sigma^{\prime }}_{c,i}+{\sigma^{\prime }}_{t,i}}\\ {\sigma^{\prime }}_{c,i}=Max\left(\sigma \left(g_{i}^{\left(c_{1}\right)},\ldots ,g_{i}^{\left(c_{\left||C|\right|}\right)}\right),0.2\times \frac{\sum_{j=1}^{\left||C|\right|}g_{i}^{\left(c_{j}\right)}}{\left||C|\right|}\right) \end{array}$$

where $g_{i}^{\left(c_{j}\right)}$ is the gene expression level for gene *g*_*i*_ in sample $A^{\left(c_{j}\right)}$ (see Figure 1); ${\sigma^{\prime }}_{c,i}$ is the standard deviation of expression levels for gene *g*_*i*_ among control samples; $g_{i}^{\left(t_{j}\right)}$ and ${\sigma^{\prime }}_{t,i}$ are defined analogously using case samples.

GSEA ranks all genes according to their scores. Then to measure the association between members of a given gene set *G*_*i*_ and treatments/phenotypes, it calculates a gene set score—also referred to as enrichment score (*ES*) in GSEA terminology—using a Kolmogorov–Smirnov statistic. The *ES* value for *G*_*i*_, denoted as *ES*(*G*_*i*_), is calculated using a running sum initialized as 0. Assume *g*_1_, …, *g*_*n*_ is the sorted list of all genes according to SNR difference in decreasing order. For each gene in the sorted list starting with the first one the running sum (enrichment score) is updated by adding a value of $+\sqrt{\frac{n-||G_{i}||}{\left||G_{i}|\right|}}$ when the gene belongs to *G*_*i*_ and by subtracting a value of $\sqrt{\frac{\left||G_{i}|\right|}{n-||G_{i}||}}$ when the gene does not belong to *G*_*i*_ (Mootha et al., 2003). The *ES* value is calculated “as the maximum observed positive deviation of the running sum” (Mootha et al., 2003), as shown in Equation (6).

(6) $$\begin{array}{l} \text{ES}\left(G_{i}\right)=\underset{1\leq l\leq n}{\max}\overset{l}{\underset{k=1}{\sum }}x_{k}\\ x_{k}=\left\{ \begin{array}{cc} +\sqrt{\frac{n-\|G_{i}\|}{\left\|G_{i}\right\|}} & R_{k}\in G_{i}\\ -\sqrt{\frac{\left\|G_{i}\right\|}{n-\|G_{i}\|}} & R_{K}\notin G_{i} \end{array}\right. \end{array}$$

After calculation of the actual *ES* values for all gene sets, the method determines the maximum *ES*, denoted as *MES*. The significance of the calculated *MES* value is assessed using a permutation test (see section 3). The sample labels are permuted 1,000 times, and for each permutation a *MES* value is calculated. Finally, the significance of *MES* of the actual data is calculated as the fraction of permutations that lead to an *MES* higher than the *MES* of the actual data.

It should be mentioned that the significance of the *MES* does not provide any insight about the significance of the enrichment score of a given gene set *G*_*i*_, although this is the main purpose of enrichment analysis. In fact, assessing the significance of the *MES* tests the null hypothesis that “no gene set is associated with the class distinction” (Mootha et al., 2003), where the rank ordering is used as the measure of association. Therefore, rejection of this null hypothesis only suggests that there is at least one gene set for which the rank ordering of its members is associated with the sample classes, i.e., phenotypes.

Since the enrichment score is defined as the “maximum observed positive deviation of the running sum” (Mootha et al., 2003), it does not detect differential enrichment of gene sets that have the majority of their genes up-regulated unless the phenotypes are swapped and the GSEA procedure is run again. Hence, this method should be considered as a one-sided test (Tian et al., 2005). In addition, in order to be able to rely on the enrichment scores, the significance of each ES should be assessed. However, the method tested the null hypothesis that “no gene set is associated with the class distinction” (Mootha et al., 2003), which is not extendable to the ES for each gene set.

Damian and Gorfine (2004) raised concerns about the capabilities of GSEA by way of suggesting a synthesized example. They showed that GSEA may ignore highly enriched gene sets solely due to the size of gene sets. In their hypothetical example they assumed that there is a given dataset of gene expression values for genes in three gene sets *G*_1_, *G*_2_, and *G*_3_ of size *n*, 5*n*, and 4*n*, respectively, where—after calculation of gene scores and sorting them—genes in *G*_1_ ranked higher than genes in *G*_2_, and genes in *G*_2_ ranked higher than genes in *G*_3_. Assume that *G*_1_ is the only enriched gene set with all genes being down-regulated, and *G*_2_ and *G*_3_ are not differentially enriched. GSEA assigns enrichment scores of 3*n*, 4*n*, and 0, respectively, to *G*_1_, *G*_2_, and *G*_3_. Therefore, *G*_2_ is preferred to *G*_1_, although *G*_1_ is the only enriched gene set. Furthermore, Subramanian et al. (Subramanian et al., 2005) reported that GSEA leads to high enrichment scores for gene sets clustered around the middle of the sorted list of all genes. These gene sets are often not associated with the phenotypes under study (Subramanian et al., 2005).

Considering these shortcomings, Tian et al. (2005) suggested using the *t*-test or Wilcoxon rank-sum test statistics as alternative gene set scores instead of the Kolmogorov–Smirnov statistic in GSEA. They suggested that these scores are able to detect moderate but coordinated shift from the background distribution. To generate the background distribution, they used both gene sampling and phenotype permutation (see section 3). In fact, instead of testing differences in distribution of gene scores across treatments, they tested a location change, i.e., shift in mean or median. The shortcoming of the method is a lack of sensitivity in detecting differentially enriched gene sets where some of its genes are up-regulated and some down-regulated (Irizarry et al., 2009). This is due to the inherent inability of the average to detect those effects.

PAGE, a parametric method for gene set enrichment analysis, was proposed as a statistically more sensitive and computationally less demanding alternative for GSEA (Kim and Volsky, 2005). PAGE tests the null hypothesis that “all genes in a given microarray dataset are independent of each other and identically distributed, that is, they are not co-regulated” (Kim and Volsky, 2005). It uses fold change between sample groups, i.e., treatments, to calculate a *Z*-score for a given gene set *G*_*i*_. The significance of this *Z*-score is then calculated using a normal distribution. PAGE starts with calculating the fold change value of each gene as the gene score. Next, it calculates mean (μ) and standard deviation (σ) of all fold change values. Then, for a given gene set *G*_*i*_, it calculates μ_*i*_ as the average fold change value of genes in *G*_*i*_. After that, a score *Z*_*i*_ is calculated as follows:

(7) $$\begin{array}{l} Z_{i}=\frac{\mu_{i}-\mu }{\frac{\sigma }{\left\|G_{i}\right\|}} \end{array}$$

Finally, the significance of *Z*_*i*_ is assessed using the standard normal distribution. The rational behind using the normal distribution is that according to the Central Limit Theorem (Freund et al., 2004), the sampling distribution of the average of an independent random variable for large sample sizes is normal, regardless of the distribution of the underlying population. Therefore, the distribution of average fold change values for gene sets should be normal. This method has been reported to achieve a high sensitivity while suffering from a low specificity (Maleki et al., 2019b).

In another attempt to address the aforementioned shortcomings of GSEA, Subramanian et al. (2005)—almost the same group who proposed GSEA—adjusted the method by using a weighted Kolmogorov–Smirnov statistic as gene set score. They also used False Discovery Rate (FDR) to adjust for multiple comparisons (Subramanian et al., 2005). First, the adjusted method calculates the gene score for each gene. Assume *g*_1_, …, *g*_*n*_ is the list of all genes sorted according to their score; then for a gene set *G*_*i*_ the gene set score is calculated as follows:

(8) $$\begin{array}{l} ES\left(G_{i}\right)=\underset{1\leq k\leq n}{max}\left(P_{hit}\left(G_{i},k\right)-P_{miss}\left(G_{i},k\right)\right)\\ P_{hit}\left(G_{i},k\right)=\underset{\underset{t\leq k}{g_{t}\in G_{i}}}{\sum }\frac{|r_{t}{|}^{p}}{R\left(G_{i}\right)}\\ R\left(G_{i}\right)=\underset{g_{t}\in G_{i}}{\sum }|r_{t}{|}^{p}\\ P_{miss}\left(G_{i},k\right)=\underset{\underset{t\leq k}{g_{t}\notin G_{i}}}{\sum }\frac{1}{n-\|G_{i}\|} \end{array}$$

where *p* is a positive constant and a parameter of the method; *r*_*t*_ is the gene score for the *t*^*th*^ gene in the sorted list. Next, the significance of the gene set scores is assessed using gene sampling or phenotype permutation (see section 3). Finally, adjustment for multiple comparisons is made.

It should be mentioned that the enrichment score in the adjusted GSEA is similar to, but not the same as, the enrichment score in GSEA. To calculate the enrichment scores, both methods calculate a running sum by traversing the list of all genes ranked according to their gene scores. For each gene in the list, the original GSEA method updates the running sum by a constant value, while the adjusted GSEA increases the running sum with a value of $\frac{|r_{t}{|}^{p}}{\underset{g_{t}\in G_{i}}{\sum }|r_{t}{|}^{p}}$ to increase the effect of genes with higher absolute value of the gene score ($|r_{t}{|}^{p}$), i.e., genes at the beginning or at the end of the ordered list, and to decrease the effect of genes in the middle. Hereafter, we use GSEA to refer to the adjusted GSEA, unless stated otherwise. GSEA is still a one-sided test. In addition, it is not obvious how GSEA addresses the effect of gene set size, as it was reported to affect the results of the original GSEA (Damian and Gorfine, 2004). Further, an *ad hoc* choice of 1 for *p* has been used in the updated version of GSEA.

Irizarry et al. (2009) proposed the use of a simple parametric method as an alternative to GSEA. They mentioned that GSEA is based on a Kolmogorov–Smirnov test which is known for its lack of sensitivity. In order to avoid using a Kolmogorov–Smirnov test statistic and also a permutation test, which is computationally demanding, they suggested using a parametric method that employs standard normal distribution to assess the significance of each enrichment score. They used the two-sample *t*-test statistic as the gene score to measure the degree of association between each gene and phenotype. For a given gene *g*, this value is denoted by *t*(*g*). They evaluated the assumption of normality of *t*(*g*) values for all genes using a Q-Q plot for 8 datasets—all datasets used by Subramanian et al. (2005) and Mootha et al. (2003). Based on the observed Q-Q plots, they suggested that assuming standard normal distribution for distribution of *t*(*g*) values in practice is valid. For a given gene set *G*_*i*_, they suggested a *Z*-score as follows:

(9) $$\begin{array}{l} \text{Z-Score}\left(G_{i}\right)=\sqrt{\left\|G_{i}\right\|}\times \bar{t}\left(G_{i}\right)\\ \bar{t}\left(G_{i}\right)=\frac{\sum_{g\in G_{i}}t\left(g\right)}{\left\|G_{i}\right\|} \end{array}$$

By accepting the assumption that the *t*-test statistic has a standard normal distribution and also ignoring the correlation between gene set members, they inferred that the *Z*-score has a standard normal distribution as well. Therefore, they assessed the significance of *Z*-scores using a standard normal distribution. Hereafter, we refer to this method as SEA.

Irizarry et al. (2009) admitted that a limitation of the proposed *Z*-score is that it may not be able to detect gene sets where almost half of the genes are up-regulated and the rest are down-regulated. To deal with this issue, they suggested a standardized χ^2^-test score as follows:

(10) $$\begin{array}{l} \chi^{2}\text{-score}\left(G_{i}\right)=\frac{\sum_{g\in G_{i}}{\left(t\left(g\right)-\bar{t}\left(G_{i}\right)\right)}^{2}-\left(\|G_{i}\|-1\right)}{2\left(\|G_{i}\|-1\right)} \end{array}$$

They approximated the distribution of the χ^2^-score for a gene set of size 20 or higher using the standard normal distribution to calculate the significance of the gene set score.

Tamayo et al. (2016) refuted the claim made by Irizarry et al. (2009) that their simple enrichment analysis method, i.e., SEA, outperforms GSEA (Subramanian et al., 2005). They focused on the assumption made by SEA to ignore gene-gene correlation, questioning its practicality and whether it is realistic. Comparing the results of SEA and GSEA, they reported that SEA uniformly produces more significant gene sets. For example, they reported that for a pancreas dataset (Abdollahi et al., 2007), SEA predicted 42% of gene sets as significantly differentially enriched, a number almost 5 times more than that from GSEA.

In addition, Tamayo et al. (2016), using the approach of Gatti et al. (2010), tested the effect of gene-gene correlation on the results of GSEA and SEA, where there was no significant correlation structure between gene profiles and phenotypes. In this regard, for each dataset, they produced results for both SEA and GSEA for 1,000 datasets resulting from the random permutation of phenotype labels in an expression profile (see section 3). Since after random permutations of gene profile labels there is almost no relation between gene profiles and phenotypes, we expect almost no significant gene set to be reported as differentially enriched by gene set enrichment analysis methods. (Tamayo et al., 2016) reported that while GSEA predicted almost 0% of gene sets as differentially enriched, SEA predicted many gene sets as differentially enriched.

Jiang and Gentleman (2007) suggested several gene and gene set scores as extensions to GSEA. They suggested a linear model for calculating a gene score. Equation (11) shows the linear model.

(11) $$\begin{array}{l} Y_{g,i}=\mu_{g}+\beta_{g}X_{i}+\epsilon_{g,i} \end{array}$$

where *Y*_*g, i*_ is the measured expression value for gene *g* from the *i*^*th*^ sample. For a given gene *g* and 1 ≤ *i* ≤ *n*, variables ϵ_*g, i*_ are assumed to be error terms that are independent and normally distributed with a mean of zero. *X*_*i*_ is a binary variable showing phenotype, i.e., class, of the *i*^*th*^ sample. For a given gene *g*, μ_*g*_ represents the mean of expression measures for the phenotype corresponding to *X*_*i*_ = 0, and β_*g*_ represents the difference between the mean of expression measures of *g* for the phenotype corresponding to *X*_*i*_ = 1 and μ_*g*_. They used $\frac{\hat{\beta_{g}}}{s_{g}}$ as the gene score, where $\hat{\beta_{g}}$ is the estimate of β and *s*_*g*_ is the estimate for standard deviation of expression measurements for gene *g*.

In addition, they suggested using median and the sign test, which is a non-parametric test to assess consistent differences in paired samples, as alternatives to the gene set statistic. The sign test was used to assess the prevalence of up- or down-regulation of genes within a gene set, regardless of the magnitude of this regulation. They found a lack of sensitivity when using the sign test as gene set score. Also, they suggested that median is less susceptible to outlier effects in comparison to using mean as a gene set score.

#### 2.2.2. Multivariate Functional Class Scoring Methods

Multivariate FCS methods, unlike single variate FCS methods, directly calculate gene set scores from expression data and skip the intermediate step of calculating gene scores (see Figure 2). Goeman et al. (2004) proposed the Globaltest method, based on a generalized linear model, to address the question whether the global expression pattern of genes in a given gene set *G*_*i*_ is significantly associated with a biological outcome of interest. The outcome of interest can be a binary group label representing two experimental conditions or a continuous variable. The idea behind the Globaltest method is that if genes in a given gene set *G*_*i*_ can be used to correctly predict a biological outcome, then genes in *G*_*i*_ should have different expression patterns for different outcomes. In Globaltest, the expression profile of genes in *G*_*i*_ across samples is represented using a matrix *X*, where *X*_*k, j*_ is the expression value of the *j*^*th*^ gene of *G*_*i*_ in the *k*^*th*^ sample; the biological outcome of interest is represented as an *n* × 1 vector *Y*, where *Y*_*k*, 1_ is the outcome of interest for the *k*^*th*^ sample. In a pairwise comparison of phenotypes, *Y*_*k*, 1_ is a binary value representing the phenotype of the *k*^*th*^ sample. In order to model the relation between *X* and *Y*, Globaltest uses the following generalized linear model:

(12) $$\begin{array}{l} E\left(Y_{i}|\beta \right)=h^{-1}\left(\alpha +\overset{m}{\underset{i=1}{\sum }}\beta_{j}x_{i,j}\right) \end{array}$$

where β_*j*_ (1 ≤ *j* ≤ *m*) is the regression coefficient for the expression value of gene *g*_*j*_; α is an intercept value; *h* is a link function. *h* can be the identity function resulting in a linear regression model, or *logit* function resulting in a logistic regression model. In order to test if genes in *G*_*i*_ are able to predict the biological outcome, the following null hypothesis should be tested.

$$\begin{array}{l} H_{0}:\beta_{1}=\beta_{2}=\cdots =\beta_{m}=0 \end{array}$$

Considering the fact that the number of samples is usually less than number of variables, i.e., gene set size ||*G*_*i*_||, this null hypothesis cannot be tested in a classical way. In order to address this problem, Goeman et al. accepted the simplifying assumption that the regression coefficients all come from the same distribution with a mean of zero and an unknown variance of τ^2^. In this case, the aforementioned null hypothesis is equivalent to the following null hypothesis:

$$\begin{array}{l} H_{0}:\tau^{2}=0 \end{array}$$

An implementation of the Globaltest method is available as an R-package from Bioconductor (Gentleman et al., 2004). The implementation uses a diagonal covariance matrix, which means that the correlation between genes in a given gene set is ignored (Ackermann and Strimmer, 2009).

Kong et al. (2006) used Hotelling's *T*^2^-test for gene set analysis. This test is the natural generalization of the *t*-test for testing the difference between multivariate means of two populations. The test statistic for a given gene set *G*_*i*_ is as follows:

(13) $$\begin{array}{l} T^{2}={\left({\bar{X}}_{C}-{\bar{X}}_{T}\right)}^{tr}{\left(S\frac{n_{1}+n_{2}}{n_{1}n_{2}}\right)}^{-1}\left({\bar{X}}_{C}-{\bar{X}}_{T}\right) \end{array}$$

where ${\bar{X}}_{C}$ and ${\bar{X}}_{T}$ are the mean expression vectors of genes in the gene set *G*_*i*_ for control and treatment samples, respectively; *n*_1_ and *n*_2_ are the number of control and treatment samples, respectively; *tr* denotes the matrix transpose operator. Under the null hypothesis (${\bar{X}}_{C}={\bar{X}}_{T}$) and when *n* > *m* + 1, the following statistic follows an F-distribution with *m* and *n* − *m* − 1 degrees of freedom, where *m* is the number of genes in *G*_*i*_ and *n* = *n*_1_ + *n*_2_:

(14) $$\begin{array}{l} \frac{n-m-1}{\left(n-2\right)m}T^{2} \end{array}$$

Since *m*, i.e., gene set size, is often bigger than *n*, i.e., sample size, Kong et al. (2006) employed single value decomposition for dimension reduction to be able to use this approach.

Successful application of multivariate statistical tests depends on meeting their stringent underlying requirements such as normality of data, adequacy of sample size, and equality of variance (Venter and Maxwell, 2000). It is almost impossible to meet all of these conditions when testing for differential enrichment of every gene set. Therefore, methods that are not robust to violating these assumptions tend to lead to irreproducible results. This has been a reason why multivariate gene set analysis methods have not been as widely used compared to univariate methods.

## 3. Significance Assessment of Gene Set Score

Based on the approach used for significance assessment, gene set analysis methods can be classified as parametric and non-parametric methods. In parametric methods, after calculating a gene set score for each gene set, a parametric distribution is used to assess the significance of this score. Non-parametric approaches, on the other hand, rely on an empirical distribution to assess the significance of the gene set scores. These methods often do not make any strong assumptions about the underlying distribution of the gene set scores. Phenotype permutation and gene sampling are the main non-parametric approaches used in gene set analysis. For example, methods such as GSEA offers both phenotype permutation and gene sampling for significance assessment.

### 3.1. Parametric Approach

The parametric approach is another way to assess the significance of gene set scores (Kim and Volsky, 2005; Irizarry et al., 2009). In this approach, first, a gene set score is proposed. Then, under the null hypothesis and by accepting some simplifying assumptions, a parametric distribution for the gene set statistic is proposed. Finally, the parametric distribution is used to assess the significance of gene set statistics.

Parametric methods are built based on some knowledge or assumptions about the underlying distribution of the gene set scores. For example, PAGE assumes that the average fold-change value of genes within a gene set follows a normal distribution. SEA, another parametric approach, assumes that its gene set score—which is a weighted average of the *t*-test score for each gene in the gene set—follows a normal distribution. Although parametric approaches are not computationally demanding, they have been criticized as being too simplistic and unable to detect truly differentially enriched gene sets (Tamayo et al., 2016).

### 3.2. Non-parametric Methods

#### 3.2.1. Gene Sampling

In gene sampling the significance of a gene set score *S*(*G*_*i*_) for a given gene set *G*_*i*_ is assessed by comparing it to the scores of randomly assembled sets of ||*G*_*i*_|| genes from the reference set *U*, i.e., all genes under study. In gene sampling method, a large number of random gene sets are assembled, and their scores are calculated. Then the significance value of the gene set score of *G*_*i*_ is calculated as the fraction of assembled gene sets that lead to stronger scores than the score of *G*_*i*_, where a score in comparison to another is considered stronger if it is more in favor of rejecting the null hypothesis of interest.

Since gene sampling does not depend on the number of samples, it has been widely used for gene set analysis of datasets with small sample sizes (Subramanian et al., 2005; Tian et al., 2005; Ackermann and Strimmer, 2009). The main shortcoming of gene sampling is that it relies on the unrealistic assumption of independence between genes within a gene set. Usually genes within a gene set show a highly correlated behavior; therefore, a gene sampling method may incorrectly predict a gene set as differentially enriched only because of high correlation between its genes. In this regard, it may cause false positive predictions. Another shortcoming of gene sampling is being computationally demanding. For each gene set *G*_*i*_, the whole process of gene set score calculation should be repeated for a large number of randomly assembled gene sets. In implementations of the gene-sampling approach, usually the number of assembled gene sets is an order of magnitude of 1,000. This number of repetitions makes the significance evaluation computationally demanding. Moreover, gene sampling may lead to a lack of statistical reliability of the significance values for large gene sets (Keller et al., 2007). Even using an order of magnitude of 1,000 assembled gene sets may not be enough to represent the background distribution; therefore, the significance value for large gene sets may not be statistically reliable.

#### 3.2.2. Phenotype Permutation

Phenotype permutation, also known as sample permutation, assesses the significance of a gene set score of a given gene set *G*_*i*_ by permuting sample labels.

First, the gene set score of *G*_*i*_ is calculated. Let *S*_*G*_*i*__ denote the gene set score of *G*_*i*_ according to the actual gene expression profile. Then a large number of expression profiles are synthesized by permuting the sample labels, i.e., the column labels of the actual expression profile. For a synthesized expression profile, we expect no association between the expression patterns of genes in *G*_*i*_ and the phenotypes. Next, for each synthesized expression profile, the gene set score of *G*_*i*_ is calculated. Finally, the significance of *S*_*G*_*i*__ is calculated as the fraction of the synthesized expression profiles that lead to a stronger score than *S*_*G*_*i*__, where a score in comparison to another is considered stronger if it is more in favor of rejecting the null hypothesis of interest.

Phenotype permutation, unlike gene sampling, does not rely on the unrealistic assumption of gene independence, but it requires a large number of samples for each phenotype. This condition most often is not satisfied. Instead, due to ethical conduct in animal and human research and limited budgets, having a large number of samples is not a choice for many researchers. In some cases, like for rare diseases, having a large sample size is not possible at all. Therefore, phenotype permutation is generally not applicable, and some gene set analysis tools provide gene sampling as an alternative to phenotype permutation (Subramanian et al., 2005).

#### 3.2.3. Dynamic Programming Approach

Keller et al. (2007) used a dynamic programming approach to assess the significance of the enrichment score used in the method proposed by Mootha et al. (2003). Their dynamic programming approach assessed the significance of the gene set scores derived from the unweighted Kolmogorov–Smirnov statistic. For a given array containing *n* genes and a given gene set *G*_*i*_, first, they calculated the gene set score *RS*_*G*_*i*__. Then they calculated its *p*-value as the probability of obtaining a gene set score equal to or greater than *RS*_*G*_*i*__, assuming that there is no association between the distribution of genes in *G*_*i*_ and the phenotypes. Since there are *n*||*G*_*i*_|| enrichment scores possible (Keller et al., 2007), they calculated the number of enrichment scores less than *RS*_*G*_*i*__ and then used the following formula to calculate the *p*-values:

(15) $$\begin{array}{l} \text{p-value}\left({\text{RS}}_{G_{i}}\right)=\\ 1-\frac{\text{number of enrichment scores that are less than }{\text{RS}}_{G_{i}}}{\left(\begin{array}{c} n\\ \left||G_{i}|\right| \end{array}\right)} \end{array}$$

In order to calculate the number of enrichment scores that are less than *RS*_*G*_*i*__ using a dynamic programming approach, they initialized a 2||*G*_*i*_||(*n* − ||*G*_*i*_|| + 1) × (*n* + 1) matrix *M*. Each row of *M*, indexed from −(*n* − ||*G*_*i*_||) × ||*G*_*i*_|| to (*n* − ||*G*_*i*_||) × ||*G*_*i*_||, represents all possible running sum scores. They initialized *M*_0, 0_ = 1 and the rest of the elements of *M* as 0. Starting from the second column (*k* = 1), they updated all elements of the matrix, column by column, according to Equation (16).

(16) $$\begin{array}{l} M\left(j,k\right)=\left\{ \begin{array}{cc} M\left(j-n+\|G_{i}\|,k-1\right)+M\left(j+\|G_{i}\|,k-1\right)\\ \text{if}-|{\text{RS}}_{G_{i}}|<j<|{\text{RS}}_{G_{i}}|\\ 0 & \text{otherwise} \end{array}\right. \end{array}$$

Finally, *M*(0, *n*) was reported as the number of enrichment scores with a maximum deviation smaller than *RS*_*G*_*i*__. Keller et al. (2007) suggested that their proposed dynamic programming approach is more efficient than the permutation approach and that their method does not suffer from the statistically unreliable results produced by the permutation method, when the number of permutations is not large enough. They claimed that their approach is almost 10 times faster than phenotype permutation and gene sampling. It should be mentioned that the main shortcoming of this approach is that, unlike permutation approach, it is not extendable to other gene set scores such as the weighted Kolmogorov–Smirnov statistic in GSEA.

## 4. Null Hypotheses in Gene Set Enrichment Analysis

**Figure 3:**
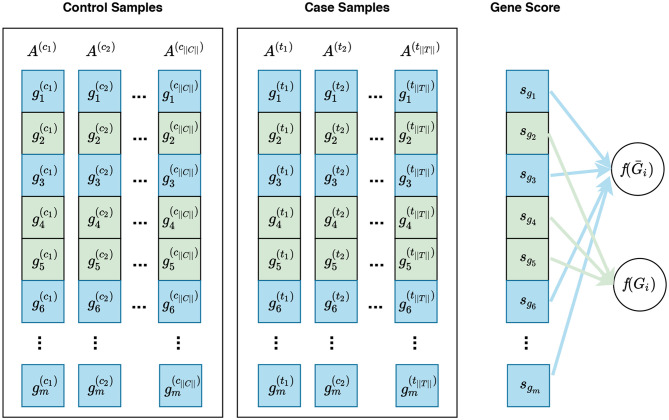
Visualization of gene sampling under the competitive null hypothesis. In this figure, $g_{i}^{\left(c_{j}\right)}$ and $g_{i}^{\left(t_{j}\right)}$ represent the expression measures for the *i*^*th*^ gene in the ${c_{j}}^{th}$ control sample and ${t_{j}}^{th}$ case sample, respectively. A competitive null hypothesis states that there is no difference between the expression patterns of genes in a given gene set in comparison to that of the rest of the genes. For example, given a gene set *G*_*i*_ consisting of three genes *G*_*i*_ = {*g*_2_, *g*_4_, *g*_5_}, depicted in green, the competitive null hypothesis states that there is no difference in the expression pattern of these genes compared to that of the rest of genes, i.e., *g*_1_, *g*_3_, *g*_6_, … , *g*_*m*_—denoted as Ḡ_*i*_ and depicted in blue. In univariate methods, for each gene *g*_*i*_, a gene score *s*_*g*_*i*__ is calculated using the expression measures for *g*_*i*_ across control and case samples. Then a gene set score *f*(*G*_*i*_)—which is representative of the difference in the expression pattern of genes in *G*_*i*_ in control samples vs. case samples—is calculated using the gene scores of genes in *G*_*i*_. Often a gene sampling approach is used for the significance assessment of the gene set score *f*(*G*_*i*_). In a multivariate setting, the intermediate step of summarizing expression values for each gene to a gene score *s*_*g*_*i*__ is omitted, and *f*(*G*_*i*_) is directly calculated from the expression values of genes in *G*_*i*_.

**Figure 4:**
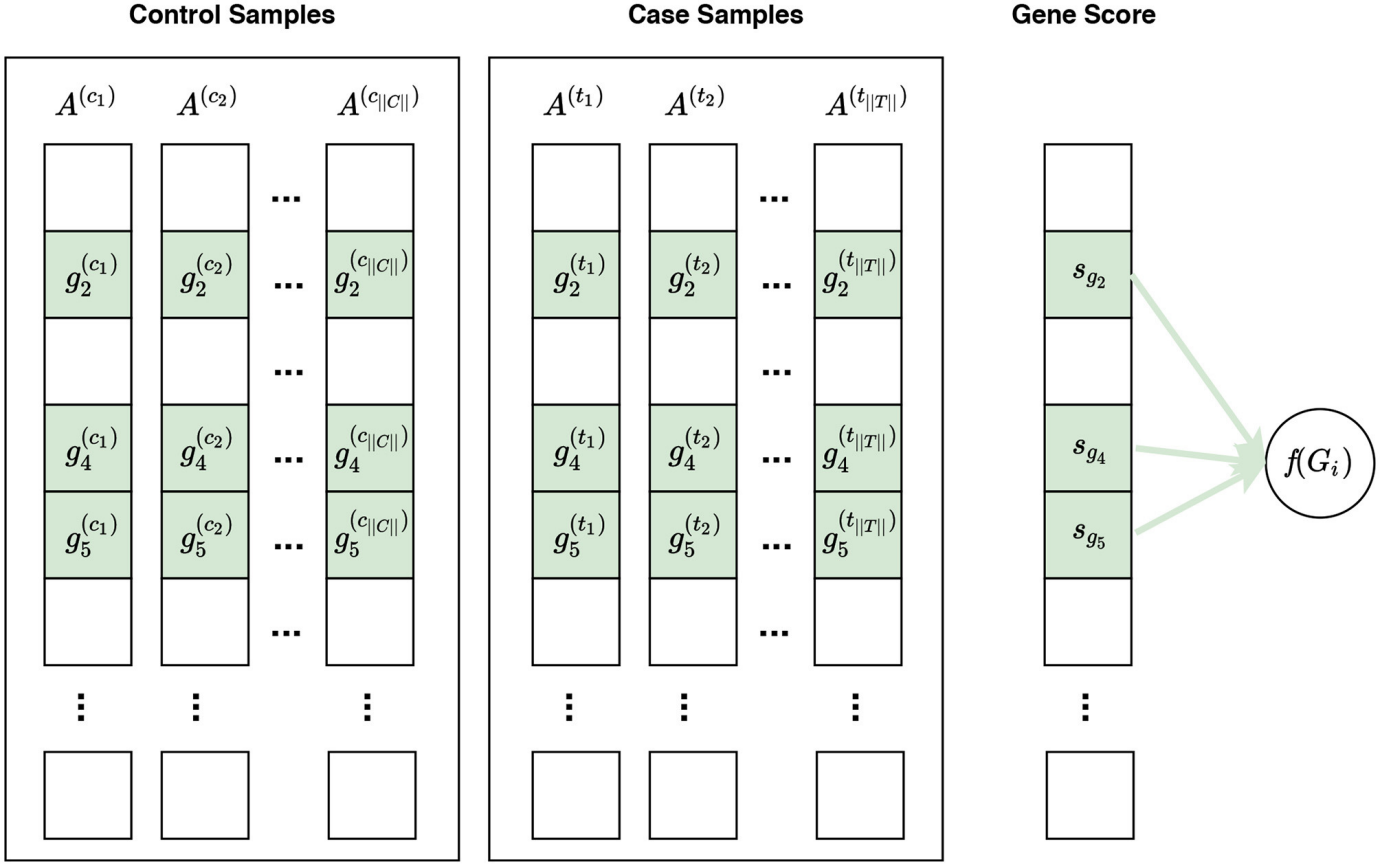
Visualization of phenotype permutation under the self-contained null hypothesis. In this figure, $g_{i}^{\left(c_{j}\right)}$ and $g_{i}^{\left(t_{j}\right)}$ represent the expression measures for the *i*^*th*^ gene in the ${c_{j}}^{th}$ control sample and ${t_{j}}^{th}$ case sample, respectively. The self-contained null hypothesis states that the expression pattern of genes within a gene set does not differ between case and control samples. For example, given a gene set *G*_*i*_ consisting of three genes *G*_*i*_ = {*g*_2_, *g*_4_, *g*_5_}, the self-contained null hypothesis states that there is no difference in the expression pattern of these genes in control samples vs. case samples. It should be noted that the self-contained null hypothesis does not concern the rest of genes, i.e., genes not in *G*_*i*_, which are shown in white here. In univariate methods, for each gene *g*_*i*_, a gene score *s*_*g*_*i*__ is calculated using the expression measures for *g*_*i*_ across control and case samples. A gene set score *f*(*G*_*i*_)—which is representative of the difference in the expression pattern of genes in *G*_*i*_ in control samples vs. case samples—is calculated using the gene scores of genes in *G*_*i*_. Often a phenotype permutation approach is used for significance assessment of the gene set score *f*(*G*_*i*_). In a multivariate setting, the intermediate step of summarizing expression values for each gene to a gene score *s*_*g*_*i*__ is omitted, and *f*(*G*_*i*_) is directly calculated from the expression values of genes in *G*_*i*_.

**Figure 5:**
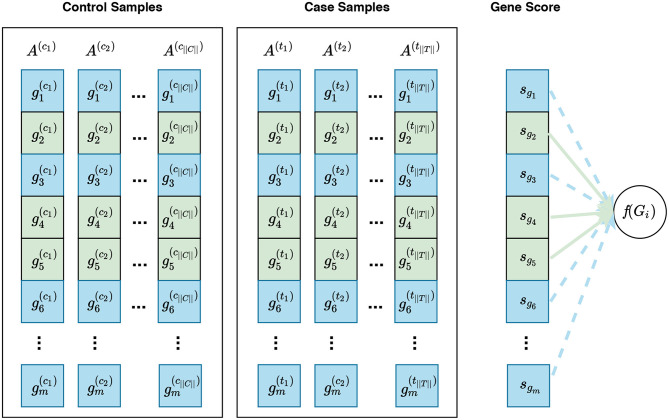
Visualization of phenotype permutation under the self-contained hybrid null hypothesis. This type of hypothesis states that the relative expression pattern of genes within a gene set is not differentially associated with phenotypes. For example, given a gene set *G*_*i*_ consisting of three genes *G*_*i*_ = {*g*_2_, *g*_4_, *g*_5_}, the hybrid null hypothesis states that there is no difference in the relative expression pattern of these genes between phenotypes. In this figure, *s*_*g*_*i*__ represents the gene score for the gene *g*_*i*_. Unlike the sample permutation approach used under a self-contained null hypothesis, not only do gene scores for genes in *G*_*i*_ contribute to the calculation of *f*(*G*_*i*_) but also gene scores for genes in Ḡ_*i*_ can contribute to this calculation. For example, the distribution of gene scores for genes in Ḡ_*i*_ can affect the enrichment score of *G*_*i*_ calculated by GSEA. The contribution of genes in *G*_*i*_ and genes in Ḡ_*i*_ are depicted with solid green lines and dashed blue lines, respectively. See Figure S1 for a visualization of gene sampling under the competitive hybrid null hypothesis.

Defining a null hypothesis is an essential step in conducting any statistical inference. Different null hypotheses have been used in gene set enrichment analysis: competitive null hypothesis (Goeman and Bühlmann, 2007), self-contained null hypothesis (Goeman and Bühlmann, 2007), and hybrid null hypothesis (Ackermann and Strimmer, 2009). Visual representations of these null hypotheses are presented in Figures 3–5, respectively. Understanding the implications of these hypotheses is essential for having a valid interpretation of the results of enrichment analysis. In this section, we discuss the limitations and requirements of each class of hypotheses.

### 4.1. Competitive Null Hypothesis

For a given gene set *G*_*i*_, a competitive null hypothesis states that genes in *G*_*i*_ do not have a different expression pattern in comparison to the rest of the genes under study (Ḡ_*i*_). Gene set analysis methods differ in the way they measures the expression pattern of genes in a gene set. Figure 3 illustrates a gene sampling approach under the competitive null hypothesis for a hypothetical gene set.

After calculation of a gene set score *f*(*G*_*i*_) for a gene set *G*_*i*_, the significance of *f*(*G*_*i*_) is assessed in an empirical manner through a gene sampling approach (see section 3.2.1). Consequently, the competitive approach has been criticized for using genes as sampling units, whereas the purpose of the experiment is to detect changes across phenotypes (Goeman and Bühlmann, 2007; Ackermann and Strimmer, 2009). It also has been criticized for ignoring the correlation between genes within a gene set. Therefore, methods based on the competitive approach may detect a gene set as being differentially enriched just because of the correlation between its genes (Goeman and Bühlmann, 2007; Ackermann and Strimmer, 2009). These methods also have been reported to be severely effected by inclusion of irrelevant genes (Tripathi et al., 2013). Consequently, different procedures used for filtering irrelevant genes lead to different statistical powers.

### 4.2. Self-Contained Null Hypothesis

For a given gene set *G*_*i*_, a self-contained null hypothesis states that genes in *G*_*i*_ do not have a different expression pattern across phenotypes. Figure 4 illustrates phenotype permutation under the self-contained null hypothesis for a hypothetical gene set.

To test the self-contained null hypothesis, a phenotype permutation approach is used (see section 3.2.2). Consequently, testing a self-contained null hypothesis leads to preserving the complex correlation of genes within a gene set. However, it requires a large number of samples for each phenotype. This condition may not be met by many biological experiments.

### 4.3. Hybrid Null Hypothesis

Hybrid null hypotheses concern changes in the relative expression patterns of genes. These null hypotheses can be classified as the competitive hybrid null hypothesis or self-contained hybrid null hypothesis. Methods based on hybrid null hypotheses calculate a gene set score for a given gene set *G*_*i*_ using expression measures from all genes, i.e., genes in *G*_*i*_ as well as genes in $\bar{G_{i}}$; then they assess the significance of this score either using a gene sampling or phenotype permutation approach. GSEA and its variants, which are based on Kolmogorov–Smirnov statistic, use hybrid null hypotheses (Mootha et al., 2003; Subramanian et al., 2005; Hung et al., 2010). For example, the current version of GSEA (version 4.0.3 which is available at www.gsea-msigdb.org) offers gene sampling for the significance assessment of a competitive hybrid null hypothesis and phenotype permutation for the significance assessment of a self-contained hybrid null hypothesis.

It should be mentioned that some authors have classified hybrid methods under competitive or self-contained methods based on whether they use a sample permutation or a gene sampling for significance assessment (Das et al., 2020). In self-contained methods, the calculated gene set score *f*(*G*_*i*_) for a gene set *G*_*i*_ is defined based on the expression values of genes in *G*_*i*_. The rest of genes, i.e., genes in Ḡ_*i*_, do not contribute to this calculation. However, in a hybrid method, genes in Ḡ_*i*_ can also contribute to the value of *f*(*G*_*i*_). Examples of hybrid methods are those using variants of Kolmogorov–Smirnov statistics, where *f*(*G*_*i*_) is defined based on the sorted list of all gene scores. Figure 4 illustrates phenotype permutation under self-contained hybrid null hypothesis for a hypothetical gene set. Figure S1 visualizes a gene sampling under the competitive hybrid null hypothesis.

## 5. Pathway Topology-Based Methods

Not all genes in a pathway play an equally important role in its activity. The knowledge of pathway topology, such as gene product interactions, can help in quantifying the importance of a gene to the pathway activity. Topology information could potentially improve the accuracy of enrichment analysis. Topology-based pathway analysis methods incorporate such information about pathways (Draghici et al., 2007; Emmert-Streib, 2007). These methods also can be classified as ORA-based, univariate, and multivariate methods. Also, they test null hypotheses similar to the manner of other gene set analysis methods (Bayerlová et al., 2015; Ihnatova et al., 2018), as described in section 4.

GSNCA was designed to account for all cross-correlations of each gene and to assign an importance value to each gene in a pathway. They compared the results of GSNCA with that of GSCA (Choi and Kendziorski, 2009). Rahmatallah et al. (2016) reported that GSNCA performed better than GSCA for large gene sets and for scenarios with a non-uniform change in the expression of pathway members.

Bayerlová et al. (2015) evaluated three competitive univariate methods—developed based on Wilcoxon rank-sum, Kolmogorov–Smirnov, and Fisher's exact test statistics—with three topology-based methods including SPIA (Tarca et al., 2009), CePa (Gu et al., 2012) (both competitive and self-contained versions), and PathNet (Dutta et al., 2012). They reported that none of the topology-based methods outperformed the univariate methods.

In a another study, Ihnatova et al. (2018) using simulated and real datasets evaluated several pathway analysis methods including: TAPPA (Gao and Wang, 2007), SPIA, TopologyGSA Massa et al. (2010), PRS (Ibrahim et al., 2012), CePa, and Clipper (Martini et al., 2013). Among these methods, TAPPA is a univariate approach; TopologyGSA and Clipper are considered multivariate methods; and SPIA, PRS, and CePa are considered ORA-based methods. They reported that the significance values reported by all of these methods correlated with pathway sizes, where large pathways achieved lower *p*-values in comparison to the smaller pathways. Also, they reported that multivariable methods—i.e., TopologyGSA and Clipper—suffered from a very low specificity, reporting a large number of false positives. In contrast, ORA-based methods—SPIA, PRS, and CePa—achieved the highest specificity.

## 6. Challenges

There are many gene set analysis methods available with no consensus about the best practices. One contributing factor to this lack of consensus is the lack of gold standard expression datasets. A gold standard dataset for evaluation of gene set analysis methods requires the enrichment status of given gene sets to be known *a priori*. The main challenges facing gene set analysis, such as the lack of reproducibility, specificity, and/or sensitivity, are rooted in the lack of gold standard datasets. If available, gold standard datasets could help with detecting and addressing these challenges.

Despite having a well-established underlying statistical model, ORA suffers from several shortcomings. ORA relies on the gene-gene independence assumption that is known to be biologically invalid (Gatti et al., 2010; Tarca et al., 2013). Also, ORA uses a list of differentially expressed genes as input and treats all genes equally regardless of their magnitude of differential expression. Moreover, differentially expressed genes are determined using a single-gene analysis method, where the use of arbitrary thresholds is often a common practice. It has been shown that the choice of these thresholds might affect the result of downstream analysis (Pan et al., 2005). ORA is also incapable of detecting low but concordant signals, i.e., below the used threshold, from genes within a gene set. These concordant signals are believed to be biologically important (Mootha et al., 2003; Subramanian et al., 2005).

FCS methods aim at solving some of these problems. There are many FCS methods available, but there is no consensus among researchers about the method of choice for a given experiment (Goeman and Bühlmann, 2007; Liu et al., 2007; Ackermann and Strimmer, 2009; Irizarry et al., 2009; Fridley et al., 2010; Hung et al., 2011; Tamayo et al., 2016; Zyla et al., 2019). Maleki et al. (2019b) proposed a systematic methodology for evaluation of 13 gene set analysis methods using real expression datasets. They showed that there is little to no overlap between the results of these methods. Also, some methods reported a large number of gene sets as being differentially enriched and some methods reported very few. This indicates that most methods either suffer from a lack of specificity (large number of false positives) or a lack of sensitivity (large number of false negatives).

Lack of specificity of gene set analysis methods is the main hindrance to gaining insight from the results of gene set analysis. For example, assume that the null hypothesis for a self-contained method is that there is no difference in the average expression of genes in a gene set between case and control samples. Then a significant change in the expression of a single gene can cause any gene set containing that gene to be reported as being differentially enriched. The problem arises in the presence of gene set overlap, where some genes may occur in several gene sets. Due to the presence of multifunctional genes (i.e., genes that play a role in several biological functions or molecular processes), and also the parent-child structure of some gene sets (e.g., gene sets extracted from GO), gene set overlap is an integral part of gene set databases (Maleki and Kusalik, 2019). However, most gene set analysis methods completely ignore this overlap. Hence, gene set overlap seems to be an important challenge that needs to be addressed. There have been several attempts to alleviate the effect of gene set overlap (Tarca et al., 2012; Simillion et al., 2017). Although these methods lead to higher specificity, they suffer from low sensitivity.

A limitation of self-contained methods is that they require a large number of samples per group, as they use phenotype permutation for significance assessment. This means that many of the high-throughput datasets available are not appropriate for use with these types of methods. Alternatively, competitive gene set analysis methods are used for datasets with small sample sizes. Competitive gene set analysis methods rely on gene sampling for the significance assessment. Gene sampling is based on the assumption that genes are independent. This assumption is known to be biologically invalid and may cause some gene sets to be predicted as being differentially enriched solely due to the correlations between its genes. This issue introduces false positives and decreases the specificity. Therefore, gene-gene correlations should be considered in the design and evaluation of gene set analysis methods.

It has been shown that for many gene set analysis methods, whether competitive or self-contained, the results of the analysis are not reproducible for small sample sizes (Maleki et al., 2019a). However, regardless of this issue, studies with small sample sizes (*n* < 5 per group) continue to be analyzed using these methods (Dumesic et al., 2019; Weinberg et al., 2019; Tan et al., 2020). Therefore, it should be stressed that the size of a dataset is an important consideration when deciding on an appropriate gene set analysis method or whether it is appropriate to use gene set analysis at all. Also, when developing new gene set analysis methods, their sensitivity to sample size should be investigated as part of the evaluation process.

Evaluation of gene set analysis methods has become an important area of research (Rahmatallah et al., 2016; Zyla et al., 2017, 2019; Mathur et al., 2018; Nguyen et al., 2019; Geistlinger et al., 2020). Gene set analysis methods have been evaluated based on real and simulated expression datasets.

Real datasets with presumed enrichment status for gene sets are commonly used for the evaluation of gene set analysis methods (Tarca et al., 2013; Zyla et al., 2017). Unfortunately, assumptions about the enrichment status of the gene sets cannot be confidently justified. Consequently, this uncertainty in the enrichment status of gene sets also leads to uncertainty in the outcome of the evaluation.

Due to the lack of gold standard datasets for the evaluation of gene set analysis methods, simulated expression datasets have been used (Efron and Tibshirani, 2007; Nam and Kim, 2008; Ackermann and Strimmer, 2009). These datasets have been developed using normally distributed expression values, with constant means and standard deviations. Also, these simulated datasets either assume no gene-gene correlation (Efron and Tibshirani, 2007; Nam and Kim, 2008) or constant correlations (Ackermann and Strimmer, 2009) between genes in gene sets. However, in practice, expression data rarely follows a normal distribution. Also, gene-gene correlation is known to be present in real expression data and has been reported to have a profound impact on the results of enrichment analysis methods (Tamayo et al., 2016). These oversimplifications might lead to evaluations that are biased in favor of some gene set analysis methods. For instance, Ackermann and Strimmer (2009) simulated expression datasets using a multivariate normal distribution with variances of 1. They simulated the expression value of non-informative genes using a standard multivariate normal distribution. They modeled differentially enriched gene sets using constant change in mean expression values and constant gene-gene correlations. Since the expression values for the non-informative genes, which comprised the majority of the dataset, followed a standard multivariate normal distribution, competitive methods and parametric methods were able to easily detect the enrichment status of gene sets. This makes the result of evaluation biased in favor of these methods. Also, normally distributed values with constant mean and standard deviation ignores heterogeneity of variance in high-throughput data (Maleki and Kusalik, 2015).

Gene set collections have also been simulated to be a small number of non-overlapping sets of equal size, a situation that is substantially different from the real gene set databases. Due to oversimplifying assumptions, evaluation of gene set analysis using these datasets has led to inconsistent and contradictory results (Maciejewski, 2013).

This is because adding unrelated genes changes the distribution of background genes. In competitive methods, the significance of a gene set score *S*(*G*_*i*_) is calculated by comparison against gene set scores derived from randomly assembled gene sets of the same size as *G*_*i*_. Adding unrelated genes increases the difference between *S*(*G*_*i*_) and the scores derived from the randomly assembled sets of genes, as unrelated genes often show a weak and non-concordant expression pattern. They also reported that GAGE, a non-parametric method, achieves a higher power when unrelated genes are added to the expression dataset. This can also be explained by the way GAGE calculates its gene set scores, which is a function of the difference between average expression values of the gene within the gene set and average expression values of the rest of the genes. By adding unrelated genes, which often show smaller average expression values, more extreme gene set scores are achieved, which in turn leads to a misleading boost in power. Tripathi et al. (2013) strongly discouraged using competitive methods such as GSEA (with gene sampling) and also GAGE.

## 7. Future Directions

Due to the lack of gold standard datasets, simulated datasets using normally distributed values with zero or constant gene-gene correlations have been widely used to evaluate gene set analysis methods (Efron and Tibshirani, 2007; Nam and Kim, 2008; Ackermann and Strimmer, 2009). Biological and technical variability alongside complex gene-gene correlation patterns cannot be modeled using such oversimplified approaches. Synthesizing datasets that preserve the true nature of gene expression data and gene set databases is an essential step in the evaluation of new and existing gene set analysis methods. More specifically, developing benchmark datasets that reflect the true nature of real datasets would be of great value for evaluation of current and new gene set analysis methods. Such a benchmark is currently absent and we suggest developing such public benchmark datasets as future research. These benchmark datasets, if publicly available, could facilitate evaluating available gene set analysis methods and facilitate developing new approaches.

One important factor that should be considered in developing gene set analysis methods is their capability in dealing with gene set overlap, which has contributed to the lack of specificity of some methods (Simillion et al., 2017; Maleki and Kusalik, 2019). Current approaches that aim at addressing gene set overlap sacrifice sensitivity and therefore introduce false negatives. Developing methods that address gene set overlap and achieve a high specificity without sacrificing sensitivity is an active research area (Tiong and Yeang, 2019; Wiebe et al., 2020) and remains as an avenue for future research.

Also, in the evaluation of gene set analysis methods, simulated gene set databases consisting of non-overlapping gene sets of equal sizes have been used. Such a setting disregards the true nature of gene set databases that have different degrees of gene set overlap and different gene set sizes, which have been reported to affect the results of gene set analysis methods (Damian and Gorfine, 2004; Simillion et al., 2017). To evaluate gene set analysis methods in a realistic context, we strongly discourage the use of such artificial gene set databases. In cases where simulated expression datasets are used, we recommend the exploration of using real gene names/IDs in the simulated expression data. This makes it possible to use real gene set databases alongside the simulated expression data. Such a small step could show the behavior of a method in addressing gene set overlap and different gene set sizes during evaluation.

Tripathi et al. (2013) showed that some competitive gene set analysis methods are sensitive to the existence of unrelated genes. When applying the competitive gene set analysis methods, we suggest following the guidelines provided by Tripathi et al. (2013). In addition, new procedures for gene set analysis should be designed to be robust against the changes in the background distribution due to the existence of unrelated genes.

Moreover, different distributions of up- and down-regulated genes in gene sets, various gene set sizes, different levels of differential expression, different sample sizes, and an imbalanced number of samples per group might affect the result of a gene set analysis method (Irizarry et al., 2009). Therefore, we suggest any attempt for evaluation or development of gene set analysis methods to consider these factors.

The quantitative study of several well-established gene set databases, which are used as input to gene set analysis methods, has shown that the choice of gene set database might have a profound impact on the results of gene set analysis (Maleki et al., 2019c). Also, genes associated with some known phenotypes are not well-represented, if at all. Therefore, regardless of the choice of gene set analysis method, gene set analysis of such phenotypes will miss those known associations. A systematic study for choosing an appropriate gene set database prior to conducting gene set analysis is another avenue for future research.

## 8. Conclusion

In this paper, we reviewed a set of well-established gene set analysis methods. We discussed the shortcomings and strengths of these methods based on their various components such as their gene set score, null hypothesis, and methods of significance assessment. We also provided direction for conducting further research in gene set analysis.

To resolve the lack of consensus about the method of choice for a given experiment, a systematic methodology for evaluating gene set analysis methods should be utilized. Developing benchmark datasets for facilitating such a method comparison would highly benefit the research community. The benchmark expression datasets should represent the characteristics of real expression data and avoid using oversimplifying assumptions such as normally distributed data with zero or constant gene-gene correlation. Also, non-overlapping genes sets of equal size must be avoided as well.

Despite the numerous gene set analysis methods and tools available, due to the complex nature of the problem, developing methods with high specificity and high sensitivity remains a challenge and an active research area.

## Author Contributions

FM wrote the first draft of the manuscript and contributed to writing revisions. KO and DH helped with writing later drafts of the manuscript, incorporating some of the recent studies in gene set analysis as well as helping with revisions. AK supervised the work and assisted with the revision of the manuscript.

## Conflict of Interest

The authors declare that the research was conducted in the absence of any commercial or financial relationships that could be construed as a potential conflict of interest.
