# Supplementary material for: Gene Set Analysis: Challenges, Opportunities, and Future Research
Source: Front Genet. 2020 Jun 30;11:654. doi: 10.3389/fgene.2020.00654 (PMC7339292; doi:10.3389/fgene.2020.00654)
Supplement: Supplementary file 1 [file Data_Sheet_1.PDF]

Supplementary Materials

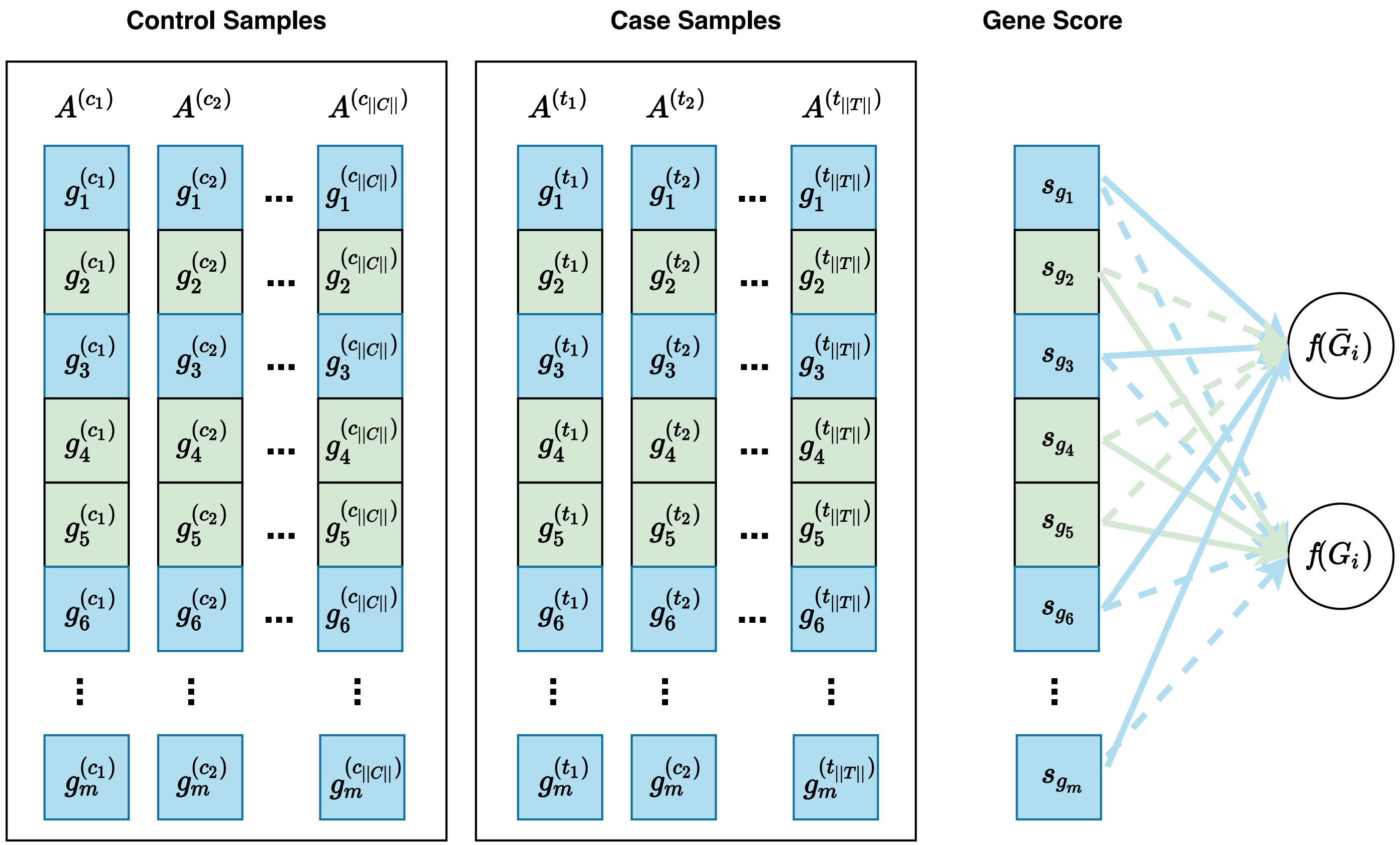

Figure S1: Visualization of gene sampling under the competitive hybrid null hypothesis. The competitive hybrid null hypothesis states that the expression pattern of genes within a gene set is not different from the expression pattern of all genes under study. For example, given a gene set G_i consisting of three genes $G_i = \{g_2, g_4, g_5\}$, the hybrid null hypothesis states that there is no difference in the expression pattern of these genes in comparison to the expression pattern of all genes. In this figure, s_{g_i} represents the gene score for the gene g_i. Note that in a hybrid method, the expression values of all genes can contribute to the calculated gene set score $f(G_i)$. The contribution of genes in G_i and genes in $\bar{G}_i$ are depicted with solid green lines and dashed blue lines, respectively. In calculation of $f(\bar{G}_i)$, contribution of genes in $\bar{G}_i$ and genes in G_i are depicted with sold blue and dashed green lines, respectively.

Table S1: Gene set analysis implementations and tools ordered by year of publication from oldest to most recent. These tools are categorized into over-representation analysis (ORA) methods, functional scoring (FCS) methods, and topology-based pathway (TP) analysis methods. Note that if a tool has been labeled as "retired", it is explicitly stated on the the website that the tool is no longer supported. All R packages are available through CRAN or Bioconductor.

Implementations/Tools	ORA	FCS	TP	Reference	Availability
Onto-Express	*			Khatri et al. (2002)	Not Available
FuncAssociate	*			Berriz et al. (2003)	http://llama.mshri.on.ca/funcassociate/
DAVID	*			Dennis et al. (2003)	https://david.ncifcrf.gov/
PANTHER	*			Thomas et al. (2003)	http://www.pantherdb.org/
GoMiner	*			Zeeberg et al. (2003)	https://discover.nci.nih.gov/gominer/index.jsp (retired)
GeneMerge	*			Castillo-Davis and Hartl (2003)	http://www.genemerge.net/ (retired)
EASE	*			Hosack et al. (2003)	https://david.ncifcrf.gov/ease/ease1.htm
Global test		*		Goeman et al. (2004)	globaltest R package
Gostat	*			Beißbarth and Speed (2004)	http://gostat.wehi.edu.au/
GOTM	*			Zhang et al. (2004)	Now merged with WebGestalt
FatiGO	*			Al-Shahrour et al. (2004)	http://www.babelomics.org
CLENCH	*			Shah and Fedoroff (2004)	https://web.stanford.edu/∼nigam/cgi-bin/dokuwiki/doku.php?id=clench#clench
GOToolBox	*			Martin et al. (2004)	https://www.webcitation.org/ (retired)
GOSurfer	*			Zhong et al. (2004)	http://systemsbio.ucsd.edu/GoSurfer/ (retired)
ScorePAGE			*	Rahnenführer et al. (2004)	Not Available
GSEA		*		Subramanian et al. (2005)	https://www.gsea-msigdb.org/gsea/index.jsp
SAFE		*		Barry et al. (2005)	safe R package
WebGestalt	*	*	*	Zhang et al. (2005)	http://www.webgestalt.org/ or R package
PAGE		*		Kim and Volsky (2005)	gage R package
ErmineJ		*		Lee et al. (2005)	https://erminej.msl.ubc.ca/
BiNGO	*			Maere et al. (2005)	Cytoscape Plugin
sigPathway		*		Tian et al. (2005)	sigpathway R package
PLAGE		*		Tomfohr et al. (2005)	GSVA R package
T-Profiler		*		Boorsma et al. (2005)	http://www.t-profiler.org/
GOTEA			*	Hu et al. (2005)	http://visant.bu.edu
WEGO	*			Ye et al. (2006)	http://wego.genomics.org.cn/
GOFFA	*			Sun et al. (2006)	http://edkb.fda.gov/webstart/arraytrack/
SAM-GS		*		Dinu et al. (2007)	Excel Ad-in
TAPPA			*	Gao and Wang (2007)	ToPASeq R package
Pathway-Express			*	Draghici et al. (2007)	Not Available
GSA		*		Efron and Tibshirani (2007)	GSA R package
Gostats	*			Falcon and Gentleman (2007)	GOstats R package
Random set		*		Newton et al. (2007)	allez R package
GOEAST	*			Zheng and Wang (2008)	Not Available
Ontologizer	*			Bauer et al. (2008)	http://ontologizer.de/

Continued on next page

Table S1 – continued from previous page

Implementations/Tools	ORA	FCS	TP	Reference	Availability
MRGSE		*		Michaud et al. (2008)	limma R package
CERNO		*		Yamaguchi et al. (2008)	http://tmod.online/
GOrilla	*			Eden et al. (2009)	http://cbl-gorilla.cs.technion.ac.il/
GAGE		*		Luo et al. (2009)	gage R package
TopGO	*			Alexa and Rahnenführer (2009)	topGO R package
SEA		*		Irizarry et al. (2009)	Not Available
SEPEA			*	Thomas et al. (2009)	Not Available
SSGSEA		*		Barbie et al. (2009)	GSVA R package
ClueGO	*			Bindea et al. (2009)	Cytoscape Plugin
MANOVA		*		Tsai and Chen (2009)	http://mail.cmu.edu.tw/∼catsai/research.htm
SPIA			*	Tarca et al. (2009)	graphite R package
ROAST		*		Wu et al. (2010)	limma R package
DECO		*		Nam (2010)	deco R package
ConceptGen	*			Sartor et al. (2010)	http://conceptgen.ncibi.org/ (retired)
TopoGSA			*	Glaab et al. (2010)	http://www.infobiotics.net/topogsa
TopologyGSA			*	Massa et al. (2010)	topologyGSA R package
PWEA			*	Hung et al. (2010)	https://zlab.bu.edu/PWEA/
NetGSA			*	Shojaie and Michailidis (2010)	netgsa R package
ADGO2	*			Chi et al. (2011)	http://www.btool.org/ADGO2
PathOlogist		*		Greenblum et al. (2011)	Not Available
NOA			*	Wang et al. (2011)	Cytoscape plugin
BPA			*	Isci et al. (2011)	http://bioinfo.unl.edu/bpa/
PADOG		*		Tarca et al. (2012)	PADOG R Package
CAMERA		*		Wu and Smyth (2012)	limma R package
goseq	*			Young et al. (2012)	goseq R package
GANPA			*	Fang et al. (2012)	GANPA R package
FunWalk			*	Komurov et al. (2012)	https://www.netwalkersuite.org/
PRS			*	Ibrahim et al. (2012)	ToPASeq R package
CePa			*	Gu et al. (2012)	CePa R package
EnrichNet			*	Glaab et al. (2012)	http://www.enrichnet.org/ (retired)
NEA			*	Alexeyenko et al. (2012)	http://fafner.meb.ki.se/personal/yudpaw/?page_id=13
ACST			*	Mieczkowski et al. (2012)	Source code available through Supplementary Material[1]
GSVA		*		Hänzelmann et al. (2013)	GSVA R package
Graphite Web			*	Sales et al. (2013)	https://graphiteweb.bio.unipd.it/
GOMA	*			Huang et al. (2013)	Not Available
Clipper			*	Martini et al. (2013)	clipper R package
Enrichr	*			Chen et al. (2013)	https://amp.pharm.mssm.edu/Enrichr/

Continued on next page

[1] https://journals.plos.org/plosone/article/file?type=supplementary&id=info:doi/10.1371/journal.pone.0041541.s001

Table S1 – continued from previous page

Implementations/Tools	ORA	FCS	TP	Reference	Availability
ROntoTools			*	Voichita and Draghici (2013)	ROntoTools R package
GSNCA		*		Rahmatallah et al. (2014, 2017)	GSAR R package
Kiwi			*	Väremo et al. (2014)	KiwiDist Python Package
mGSZ		*		Mishra et al. (2014)	mGSZ R package
PAEA		*		Clark et al. (2015)	http://amp.pharm.mssm.edu/PAEA/
ToPASeq			*	Ihnatova and Budinska (2015)	ToPASeq R package
FRY		*		Giner and Smyth (2016)	limma R package
TcGSA		*		Hejblum et al. (2015)	TcGSA R package
SGSE		*		Frost et al. (2015)	PCGSE R package
DRAGEN			*	Ma et al. (2015)	http://bioinfo.au.tsinghua.edu.cn/dragen/
Weighted-SAMGSR			*	Tian et al. (2016)	Source code available through Supplementary Material[2]
LEGO	*			Dong et al. (2016)	https://github.com/SherryDong/LEGO
Cogena		*		Jia et al. (2016)	cogena R package
GeneAnalytics	*			Ben-Ari Fuchs et al. (2016)	https://geneanalytics.genecards.org/
g:Profiler	*			Reimand et al. (2016)	https://biit.cs.ut.ee/gprofiler/gost
EnrichmentBrowser			*	Geistlinger et al. (2016)	EnrichmentBrowser R package
IGSA		*		Wu et al. (2016)	http://210.46.85.180:8080/IGSAWeb/
BLMA			*	Nguyen and Draghici (2017)	BLMA R package
SetRAnk		*		Simillion et al. (2017)	SetRank R package
Funnel-GSEA		*		Zhang et al. (2017)	https://github.com/yunzhang813/FUNNEL-GSEA-R-Package
EGSEA		*		Alhamdoosh et al. (2017)	EGSEA R package
GSAQ	*			Das et al. (2018)	GSAQ R package
singscore		*		Foroutan et al. (2018)	singscore R package
ShinnyGO	*			Ge and Jung (2018)	http://bioinformatics.sdstate.edu/go/
SCIA			*	Li et al. (2019)	https://github.com/YiqunLiHIT/SCIA
MGSEA		*		Tiong and Yeang (2019)	Source code available through Supplementary Material[3]
GSEPD		*		Stamm et al. (2019)	rgsepd R package
FunSet	*			Hale et al. (2019)	http://funset.uno
gwSPIA			*	Bao et al. (2019)	gwSPIA R package

References

Al-Shahrour, F., Díaz-Uriarte, R., and Dopazo, J. (2004). FatiGO: a web tool for finding significant associations of gene ontology terms with groups of genes. *Bioinformatics* 20, 578–580

Alexa, A. and Rahnenführer, J. (2009). Gene set enrichment analysis with topGO. *Bioconductor Improv* 27

[2]https://static-content.springer.com/esm/art%3A10.1186%2Fs13062-016-0152-3/MediaObjects/13062_2016_152_MOESM1_ESM.docx
[3]https://static-content.springer.com/esm/art%3A10.1186%2Fs12859-019-2716-6/MediaObjects/12859_2019_2716_MOESM20_ESM.zip

Alexeyenko, A., Lee, W., Pernemalm, M., Guegan, J., Dessen, P., Lazar, V., et al. (2012). Network enrichment analysis: extension of gene-set enrichment analysis to gene networks. *BMC Bioinformatics* 13, 226

Alhamdoosh, M., Ng, M., Wilson, N. J., Sheridan, J. M., Huynh, H., Wilson, M. J., et al. (2017). Combining multiple tools outperforms individual methods in gene set enrichment analyses. *Bioinformatics* 33, 414–424

Bao, Z., Zhu, Y., Ge, Q., Gu, W., Dong, X., and Bai, Y. (2019). gwSPIA: Improved signaling pathway impact analysis with gene weights. *IEEE Access* 7, 69172–69183

Barbie, D. A., Tamayo, P., Boehm, J. S., Kim, S. Y., Moody, S. E., Dunn, I. F., et al. (2009). Systematic rna interference reveals that oncogenic kras-driven cancers require tbk1. *Nature* 462, 108

Barry, W. T., Nobel, A. B., and Wright, F. A. (2005). Significance analysis of functional categories in gene expression studies: a structured permutation approach. *Bioinformatics* 21, 1943–1949

Bauer, S., Grossmann, S., Vingron, M., and Robinson, P. N. (2008). Ontologizer 2.0a multifunctional tool for GO term enrichment analysis and data exploration. *Bioinformatics* 24, 1650–1651

Beißbarth, T. and Speed, T. P. (2004). GOstat: find statistically overrepresented gene ontologies within a group of genes. *Bioinformatics* 20, 1464–1465

Ben-Ari Fuchs, S., Lieder, I., Stelzer, G., Mazor, Y., Buzhor, E., Kaplan, S., et al. (2016). GeneAnalytics: an integrative gene set analysis tool for next generation sequencing, RNA-seq and microarray data. *Omics: A Journal of Integrative Biology* 20, 139–151

Berriz, G. F., King, O. D., Bryant, B., Sander, C., and Roth, F. P. (2003). Characterizing gene sets with funcassociate. *Bioinformatics* 19, 2502–2504

Bindea, G., Mlecnik, B., Hackl, H., Charoentong, P., Tosolini, M., Kirilovsky, A., et al. (2009). ClueGO: a Cytoscape plug-in to decipher functionally grouped gene ontology and pathway annotation networks. *Bioinformatics* 25, 1091–1093

Boorsma, A., Foat, B. C., Vis, D., Klis, F., and Bussemaker, H. J. (2005). T-profiler: scoring the activity of predefined groups of genes using gene expression data. *Nucleic Acids Research* 33, W592–W595

Castillo-Davis, C. I. and Hartl, D. L. (2003). GeneMerge—post-genomic analysis, data mining, and hypothesis testing. *Bioinformatics* 19, 891–892

Chen, E. Y., Tan, C. M., Kou, Y., Duan, Q., Wang, Z., Meirelles, G. V., et al. (2013). Enrichr: interactive and collaborative HTML5 gene list enrichment analysis tool. *BMC Bioinformatics* 14, 128

Chi, S.-M., Kim, J., Kim, S.-Y., and Nam, D. (2011). ADGO 2.0: interpreting microarray data and list of genes using composite annotations. *Nucleic Acids Research* 39, W302–W306

Clark, N. R., Szymkiewicz, M., Wang, Z., Monteiro, C. D., Jones, M. R., and Ma'ayan, A. (2015). Principle angle enrichment analysis (PAEA): Dimensionally reduced multivariate gene set enrichment analysis tool. In *2015 IEEE International Conference on Bioinformatics and Biomedicine (BIBM)* (IEEE), 256–262

Das, S., Rai, A., Mishra, D. C., and Rai, S. N. (2018). Statistical approach for gene set analysis with trait specific quantitative trait loci. *Scientific Reports* 8, 1–12

Dennis, G., Sherman, B. T., Hosack, D. A., Yang, J., Gao, W., Lane, H. C., et al. (2003). DAVID: database for annotation, visualization, and integrated discovery. *Genome Biology* 4, R60

Dinu, I., Potter, J. D., Mueller, T., Liu, Q., Adewale, A. J., Jhangri, G. S., et al. (2007). Improving gene set analysis of microarray data by sam-gs. *BMC Bioinformatics* 8, 242

Dong, X., Hao, Y., Wang, X., and Tian, W. (2016). LEGO: a novel method for gene set over-representation analysis by incorporating network-based gene weights. *Scientific Reports* 6, 18871

Draghici, S., Khatri, P., Tarca, A. L., Amin, K., Done, A., Voichita, C., et al. (2007). A systems biology approach for pathway level analysis. *Genome Research* 17, 1537–1545

Eden, E., Navon, R., Steinfeld, I., Lipson, D., and Yakhini, Z. (2009). GOrilla: a tool for discovery and visualization of enriched GO terms in ranked gene lists. *BMC Bioinformatics* 10, 48

Efron, B. and Tibshirani, R. (2007). On testing the significance of sets of genes. *The Annals of Applied Statistics* , 107–129

Falcon, S. and Gentleman, R. (2007). Using GOstats to test gene lists for GO term association. *Bioinformatics* 23, 257–258

Fang, Z., Tian, W., and Ji, H. (2012). A network-based gene-weighting approach for pathway analysis. *Cell Research* 22, 565–580

Foroutan, M., Bhuva, D. D., Lyu, R., Horan, K., Cursons, J., and Davis, M. J. (2018). Single sample scoring of molecular phenotypes. *BMC Bioinformatics* 19, 1–10

Frost, H. R., Li, Z., and Moore, J. H. (2015). Spectral gene set enrichment (SGSE). *BMC Bioinformatics* 16, 70

Gao, S. and Wang, X. (2007). Tappa: topological analysis of pathway phenotype association. *Bioinformatics* 23, 3100–3102

Ge, S. and Jung, D. (2018). ShinyGO: a graphical enrichment tool for animals and plants. *Biorxiv* , 315150

Geistlinger, L., Csaba, G., and Zimmer, R. (2016). Bioconductors EnrichmentBrowser: seamless navigation through combined results of set-& network-based enrichment analysis. *BMC Bioinformatics* 17, 45

Giner, G. and Smyth, G. K. (2016). FRY: a fast approximation to ROAST gene set test with mean aggregated set statistics. *F1000Research* 5

Glaab, E., Baudot, A., Krasnogor, N., Schneider, R., and Valencia, A. (2012). EnrichNet: network-based gene set enrichment analysis. *Bioinformatics* 28, i451–i457

Glaab, E., Baudot, A., Krasnogor, N., and Valencia, A. (2010). TopoGSA: network topological gene set analysis. *Bioinformatics* 26, 1271–1272

Goeman, J. J., Van De Geer, S. A., De Kort, F., and Van Houwelingen, H. C. (2004). A global test for groups of genes: testing association with a clinical outcome. *Bioinformatics* 20, 93–99

Greenblum, S. I., Efroni, S., Schaefer, C. F., and Buetow, K. H. (2011). The PathOlogist: an automated tool for pathway-centric analysis. *BMC Bioinformatics* 12, 133

Gu, Z., Liu, J., Cao, K., Zhang, J., and Wang, J. (2012). Centrality-based pathway enrichment: a systematic approach for finding significant pathways dominated by key genes. *BMC systems biology* 6, 56

Hale, M. L., Thapa, I., and Ghersi, D. (2019). FunSet: an open-source software and web server for performing and displaying gene ontology enrichment analysis. *BMC Bioinformatics* 20, 359

Hänzelmann, S., Castelo, R., and Guinney, J. (2013). Gsva: gene set variation analysis for microarray and RNA-Seq data. *BMC Bioinformatics* 14, 7

Hejblum, B. P., Skinner, J., and Thiébaut, R. (2015). Time-course gene set analysis for longitudinal gene expression data. *PLoS Computational Biology* 11

Hosack, D. A., Dennis, G., Sherman, B. T., Lane, H. C., and Lempicki, R. A. (2003). Identifying biological themes within lists of genes with EASE. *Genome Biology* 4, R70

Hu, Z., Mellor, J., Wu, J., Yamada, T., Holloway, D., and DeLisi, C. (2005). VisANT: data-integrating visual framework for biological networks and modules. *Nucleic Acids Research* 33, W352–W357

Huang, Q., Wu, L.-Y., Wang, Y., and Zhang, X.-S. (2013). GOMA: functional enrichment analysis tool based on GO modules. *Chinese Journal of Cancer* 32, 195

Hung, J.-H., Whitfield, T. W., Yang, T.-H., Hu, Z., Weng, Z., DeLisi, C., et al. (2010). Identification of functional modules that correlate with phenotypic difference: the influence of network topology. *Genome Biology* 11, R23

Ibrahim, M. A.-H., Jassim, S., Cawthorne, M. A., and Langlands, K. (2012). A topology-based score for pathway enrichment. *Journal of Computational Biology* 19, 563–573

Ihnatova, I. and Budinska, E. (2015). ToPASeq: an R package for topology-based pathway analysis of microarray and RNA-seq data. *BMC Bioinformatics* 16, 350

Irizarry, R. A., Wang, C., Zhou, Y., and Speed, T. P. (2009). Gene set enrichment analysis made simple. *Statistical Methods in Medical Research* 18, 565–575

Isci, S., Ozturk, C., Jones, J., and Otu, H. H. (2011). Pathway analysis of high-throughput biological data within a bayesian network framework. *Bioinformatics* 27, 1667–1674

Jia, Z., Liu, Y., Guan, N., Bo, X., Luo, Z., and Barnes, M. R. (2016). Cogena, a novel tool for co-expressed gene-set enrichment analysis, applied to drug repositioning and drug mode of action discovery. *BMC Genomics* 17, 414

Khatri, P., Draghici, S., Ostermeier, G. C., and Krawetz, S. A. (2002). Profiling gene expression using onto-express. *Genomics* 79, 266–270

Kim, S.-Y. and Volsky, D. J. (2005). PAGE: parametric analysis of gene set enrichment. *BMC Bioinformatics* 6, 144

Komurov, K., Dursun, S., Erdin, S., and Ram, P. T. (2012). NetWalker: a contextual network analysis tool for functional genomics. *BMC Genomics* 13, 282

Lee, H. K., Braynen, W., Keshav, K., and Pavlidis, P. (2005). Erminej: tool for functional analysis of gene expression data sets. *BMC Bioinformatics* 6, 269

Li, Y., Wu, Y., Zhang, X., Bai, Y., Akthar, L. M., Lu, X., et al. (2019). SCIA: A novel gene set analysis applicable to data with different characteristics. *Frontiers in Genetics* 10

Luo, W., Friedman, M. S., Shedden, K., Hankenson, K. D., and Woolf, P. J. (2009). Gage: generally applicable gene set enrichment for pathway analysis. *BMC Bioinformatics* 10, 161

Ma, S., Jiang, T., and Jiang, R. (2015). Differential regulation enrichment analysis via the integration of transcriptional regulatory network and gene expression data. *Bioinformatics* 31, 563–571

Maere, S., Heymans, K., and Kuiper, M. (2005). BiNGO: a cytoscape plugin to assess overrepresentation of gene ontology categories in biological networks. *Bioinformatics* 21, 3448–3449

Martin, D., Brun, C., Remy, E., Mouren, P., Thieffry, D., and Jacq, B. (2004). GOToolBox: functional analysis of gene datasets based on gene ontology. *Genome Biology* 5, R101

Martini, P., Sales, G., Massa, M. S., Chiogna, M., and Romualdi, C. (2013). Along signal paths: an empirical gene set approach exploiting pathway topology. *Nucleic Acids Research* 41, e19–e19

Massa, M. S., Chiogna, M., and Romualdi, C. (2010). Gene set analysis exploiting the topology of a pathway. *BMC Systems Biology* 4, 121

Michaud, J., Simpson, K. M., Escher, R., Buchet-Poyau, K., Beissbarth, T., Carmichael, C., et al. (2008). Integrative analysis of runx1 downstream pathways and target genes. *BMC Genomics* 9, 363

Mieczkowski, J., Swiatek-Machado, K., and Kaminska, B. (2012). Identification of pathway deregulation–gene expression based analysis of consistent signal transduction. *PloS One* 7

Mishra, P., Törönen, P., Leino, Y., and Holm, L. (2014). Gene set analysis: limitations in popular existing methods and proposed improvements. *Bioinformatics* 30, 2747–2756

Nam, D. (2010). De-correlating expression in gene-set analysis. *Bioinformatics* 26, i511–i516

Newton, M. A., Quintana, F. A., Den Boon, J. A., Sengupta, S., and Ahlquist, P. (2007). Random-set methods identify distinct aspects of the enrichment signal in gene-set analysis. *The Annals of Applied Statistics* , 85–106

Nguyen, T. and Draghici, S. (2017). BLMA: A package for bi-level meta-analysis. *Bioconductor. R package*

Rahmatallah, Y., Emmert-Streib, F., and Glazko, G. (2014). Gene sets net correlations analysis (GSNCA): a multivariate differential coexpression test for gene sets. *Bioinformatics* 30, 360–368

Rahmatallah, Y., Zybailov, B., Emmert-Streib, F., and Glazko, G. (2017). GSAR: bioconductor package for gene set analysis in r. *BMC Bioinformatics* 18, 61

Rahnenführer, J., Domingues, F. S., Maydt, J., and Lengauer, T. (2004). Calculating the statistical significance of changes in pathway activity from gene expression data. *Statistical Applications in Genetics and Molecular Biology* 3

Reimand, J., Arak, T., Adler, P., Kolberg, L., Reisberg, S., Peterson, H., et al. (2016). g:profilera web server for functional interpretation of gene lists (2016 update). *Nucleic Acids Research* 44, W83–W89

Sales, G., Calura, E., Martini, P., and Romualdi, C. (2013). Graphite Web: Web tool for gene set analysis exploiting pathway topology. *Nucleic Acids Research* 41, W89–W97

Sartor, M. A., Mahavisno, V., Keshamouni, V. G., Cavalcoli, J., Wright, Z., Karnovsky, A., et al. (2010). ConceptGen: a gene set enrichment and gene set relation mapping tool. *Bioinformatics* 26, 456–463

Shah, N. and Fedoroff, N. V. (2004). CLENCH: a program for calculating cluster enrichment using the gene ontology. *Bioinformatics* 20, 1196–1197

Shojaie, A. and Michailidis, G. (2010). Network enrichment analysis in complex experiments. *Statistical Applications in Genetics and Molecular Biology* 9

Simillion, C., Liechti, R., Lischer, H. E., Ioannidis, V., and Bruggmann, R. (2017). Avoiding the pitfalls of gene set enrichment analysis with setrank. *BMC Bioinformatics* 18, 151

Stamm, K., Tomita-Mitchell, A., and Bozdag, S. (2019). GSEPD: a bioconductor package for RNA-seq gene set enrichment and projection display. *BMC Bioinformatics* 20, 115

Subramanian, A., Tamayo, P., Mootha, V. K., Mukherjee, S., Ebert, B. L., Gillette, M. A., et al. (2005). Gene set enrichment analysis: a knowledge-based approach for interpreting genome-wide expression profiles. *Proceedings of the National Academy of Sciences of the United States of America* 102, 15545–15550

Sun, H., Fang, H., Chen, T., Perkins, R., and Tong, W. (2006). GOFFA: gene ontology for functional analysis–a FDA gene ontology tool for analysis of genomic and proteomic data. In *BMC Bioinformatics* (BioMed Central), vol. 7, S23

Tarca, A. L., Draghici, S., Bhatti, G., and Romero, R. (2012). Down-weighting overlapping genes improves gene set analysis. *BMC Bioinformatics* 13, 136

Tarca, A. L., Draghici, S., Khatri, P., Hassan, S. S., Mittal, P., Kim, J.-s., et al. (2009). A novel signaling pathway impact analysis. *Bioinformatics* 25, 75–82

Thomas, P. D., Campbell, M. J., Kejariwal, A., Mi, H., Karlak, B., Daverman, R., et al. (2003). PANTHER: a library of protein families and subfamilies indexed by function. *Genome Research* 13, 2129–2141

Thomas, R., Gohlke, J. M., Stopper, G. F., Parham, F. M., and Portier, C. J. (2009). Choosing the right path: enhancement of biologically relevant sets of genes or proteins using pathway structure. *Genome Biology* 10, R44

Tian, L., Greenberg, S. A., Kong, S. W., Altschuler, J., Kohane, I. S., and Park, P. J. (2005). Discovering statistically significant pathways in expression profiling studies. *Proceedings of the National Academy of Sciences of the United States of America* 102, 13544–13549

Tian, S., Chang, H. H., and Wang, C. (2016). Weighted-SAMGSR: combining significance analysis of microarray-gene set reduction algorithm with pathway topology-based weights to select relevant genes. *Biology Direct* 11, 50

Tiong, K.-L. and Yeang, C.-H. (2019). MGSEA–a multivariate gene set enrichment analysis. *BMC Bioinformatics* 20, 145

Tomfohr, J., Lu, J., and Kepler, T. B. (2005). Pathway level analysis of gene expression using singular value decomposition. *BMC Bioinformatics* 6, 225

Tsai, C.-A. and Chen, J. J. (2009). Multivariate analysis of variance test for gene set analysis. *Bioinformatics* 25, 897–903

Väremo, L., Gatto, F., and Nielsen, J. (2014). Kiwi: a tool for integration and visualization of network topology and gene-set analysis. *BMC Bioinformatics* 15, 408

Voichita, C. and Draghici, S. (2013). ROntoTools: The r Onto-Tools suite. *R package version* 1

Wang, J., Huang, Q., Liu, Z.-P., Wang, Y., Wu, L.-Y., Chen, L., et al. (2011). NOA: a novel network ontology analysis method. *Nucleic Acids Research* 39, e87–e87

Wu, D., Lim, E., Vaillant, F., Asselin-Labat, M.-L., Visvader, J. E., and Smyth, G. K. (2010). ROAST: rotation gene set tests for complex microarray experiments. *Bioinformatics* 26, 2176–2182

Wu, D. and Smyth, G. K. (2012). Camera: a competitive gene set test accounting for inter-gene correlation. *Nucleic Acids Research* 40, e133–e133

Wu, L., Chen, X., Zhang, D., Zhang, W., Liu, L., Ma, H., et al. (2016). IGSA: Individual gene sets analysis, including enrichment and clustering. *PloS One* 11

Yamaguchi, K. D., Ruderman, D. L., Croze, E., Wagner, T. C., Velichko, S., Reder, A. T., et al. (2008). IFN-β-regulated genes show abnormal expression in therapy-naïve relapsing–remitting MS mononuclear cells: Gene expression analysis employing all reported protein–protein interactions. *Journal of Neuroimmunology* 195, 116–120

Ye, J., Fang, L., Zheng, H., Zhang, Y., Chen, J., Zhang, Z., et al. (2006). WEGO: a web tool for plotting GO annotations. *Nucleic Acids Research* 34, W293–W297

Young, M. D., Wakefield, M. J., Smyth, G. K., and Oshlack, A. (2012). goseq: Gene ontology testing for RNA-seq datasets. *R Bioconductor* 8, 1–25

Zeeberg, B. R., Feng, W., Wang, G., Wang, M. D., Fojo, A. T., Sunshine, M., et al. (2003). GoMiner: a resource for biological interpretation of genomic and proteomic data. *Genome Biology* 4, R28

Zhang, B., Kirov, S., and Snoddy, J. (2005). WebGestalt: an integrated system for exploring gene sets in various biological contexts. *Nucleic Acids Research* 33, W741–W748

Zhang, B., Schmoyer, D., Kirov, S., and Snoddy, J. (2004). GOTree Machine (GOTM): a web-based platform for interpreting sets of interesting genes using gene ontology hierarchies. *BMC Bioinformatics* 5, 16

Zhang, Y., Topham, D. J., Thakar, J., and Qiu, X. (2017). FUNNEL-GSEA: FUNctioNal ELastic-netregression in time-course gene set enrichment analysis. *Bioinformatics* 33, 1944–1952

Zheng, Q. and Wang, X.-J. (2008). GOEAST: a web-based software toolkit for gene ontology enrichment analysis. *Nucleic Acids Research* 36, W358–W363

Zhong, S., Storch, K.-F., Lipan, O., Kao, M.-C. J., Weitz, C. J., and Wong, W. H. (2004). GoSurfer. *Applied Bioinformatics* 3, 261–264
